# The L108I polymorphism in mouse prion protein drives spontaneous disease and enhances transmission of atypical and classical prion strains

**DOI:** 10.1111/bpa.70083

**Published:** 2026-02-09

**Authors:** Hasier Eraña, Enric Vidal, Natalia Fernández‐Borges, Jorge M. Charco, Carlos M. Díaz‐Domínguez, Cristina Sampedro‐Torres‐Quevedo, Josu Galarza‐Ahumada, Eva Fernández‐Muñoz, Maitena San‐Juan‐Ansoleaga, Miguel Ángel Pérez‐Castro, Nuno Gonçalves‐Anjo, Patricia Piñeiro, Samanta Giler, Nora González‐Martín, Nuria L. Lorenzo, Africa Manero‐Azua, Guiomar Perez de Nanclares, Mariví Geijo, Manuel A. Sánchez‐Martín, Jesús R. Requena, Joaquín Castilla

**Affiliations:** ^1^ Center for Cooperative Research in Biosciences (CIC BioGUNE) Basque Research and Technology Alliance (BRTA) Derio Spain; ^2^ Centro de Investigación Biomédica en Red de Enfermedades infecciosas (CIBERINFEC) Carlos III National Health Institute Madrid Spain; ^3^ ATLAS Molecular Pharma S. L. Derio Spain; ^4^ IRTA, Programa de Sanitat Animal Centre de Recerca en Sanitat Animal (CReSA), Campus de la Universitat Autònoma de Barcelona (UAB) Bellaterra Catalonia Spain; ^5^ Unitat mixta d'Investigació IRTA‐UAB en Sanitat Animal Centre de Recerca en Sanitat Animal (CReSA), Campus de la Universitat Autònoma de Barcelona (UAB) Bellaterra Catalonia Spain; ^6^ Institute for Biomedical Research of Salamanca (IBSAL) Salamanca Spain; ^7^ CIMUS Biomedical Research Institute University of Santiago de Compostela‐IDIS Santiago Spain; ^8^ Molecular (Epi)Genetics Laboratory Bioaraba Health Research Institute, Araba University Hospital Vitoria‐Gasteiz Spain; ^9^ Animal Health Department NEIKER‐Basque Institute for Agricultural Research and Development, Basque Research and Technology Alliance (BRTA) Derio Spain; ^10^ Transgenic Facility, Department of Medicine University of Salamanca Salamanca Spain; ^11^ IKERBASQUE Basque Foundation for Science Bilbao Spain

**Keywords:** atypical prions, CRISPR‐Cas, L108I polymorphism, prion diseases, prion strains, prion transmission, spontaneous neurodegeneration, transgenic mice

## Abstract

Prion diseases are fatal neurodegenerative disorders that can be idiopathic, associated with genetic mutations, or acquired by infection with misfolded prion protein. We developed two complementary transgenic mouse models to investigate how the L108I substitution in mouse prion protein (PrP) influences spontaneous prion formation and transmission characteristics. The transgenic mouse model overexpressing the variant at approximately three times wild‐type (WT) PrP levels (TgMo(L108I)3x) consistently developed a spontaneous neurodegenerative disorder between 219 and 536 days of age with 100% penetrance. This spontaneous disease exhibited biochemical and neuropathological characteristics of atypical prion disorders, featuring a distinctive 7–10 kDa protease‐resistant PrP fragment and pathology comparable to small ruminants' atypical scrapie and certain forms of Gerstmann–Sträussler–Scheinker syndrome (GSS). In contrast, the knock‐in model expressing the same variant at physiological levels (TgMo(L108I)1x) showed no spontaneous disease beyond 600 days, demonstrating that both the specific amino acid substitution and elevated expression levels are necessary for spontaneous prion formation. The spontaneously generated prions transmitted efficiently to models expressing the I108 variant and to Tga20 mice overexpressing WT PrP but encountered a robust transmission barrier toward WT mice, indicating strain‐specific replication requirements. The TgMo(L108I)3x model demonstrated exceptional versatility as a universal acceptor for heterogeneous prion isolates, demonstrating superior efficiency in propagating atypical variants like GSS A117V (57 ± 0.6 days) and rapid propagation of classical scrapie‐derived mouse prion strains, including Rocky Mountains Laboratory mouse prion strain (RML) (85 ± 3.8 days) and 22L (95 ± 1 days). Comparative analysis revealed that the L108I substitution differentially impacts strain propagation, with greater acceleration of RML (~33% shorter incubation) than 22L (~0.5% shorter) compared to WT mice. These complementary systems offer powerful experimental platforms for investigating the molecular determinants of spontaneous prion formation, strain selection and transmission barriers, providing insights into idiopathic prion pathogenesis and developing therapeutic interventions.

## Graphical abstract

A single amino acid change (L108I) combined with PrP overexpression drives spontaneous atypical prion formation in mice, enabling also efficient propagation of diverse prion strains. This model allows studying how spontaneous prion diseases arise and provides powerful tools for investigating strain emergence, transmission barriers, and mechanisms relevant to human neurodegenerative disorders.

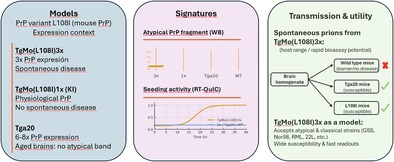

## 1 INTRODUCTION

The conversion of the cellular prion protein (PrP^C^) to its pathogenic, misfolded form (PrP^Sc^) defines prion diseases, a group of invariably fatal neurodegenerative disorders [1]. Research into these conditions has progressed significantly through the creation of diverse transgenic mouse models, each contributing valuable insights into transmission dynamics and disease mechanisms. Early studies with wild‐type (WT) mice carrying different PrP genotypes laid important groundwork [2], subsequently enhanced by studies using mice with targeted genetic modifications [3]. Despite these advances, current models fall short of capturing spontaneous prion formation that fully mimics natural disease processes. Most notably, we lack models that spontaneously generate classical prion strains characterized by the signature three‐band pattern of protease K‐resistant fragments and efficient transmissibility to WT hosts—key features for studying disease mechanisms accurately.

While perfect models remain elusive, researchers have created systems that partially recapitulate spontaneous prion formation. Interestingly, these experimental models primarily generate “atypical” prion conformers rather than classical strains [4, 5, 6, 7]. This distinction mirrors natural prion diseases, which occur spontaneously in sheep, goats, deer, cattle, and humans [8]. The atypical idiopathic forms are most clearly defined in sheep with Nor98, characterized by a distinctive ~12 kDa protease‐resistant fragment and primarily cortical rather than brainstem pathology [9], and in humans with variably protease sensitive prionotpathy, which shows multiple proteinase K (PK)‐resistant PrP fragments in a ladder pattern distinctly different from typical Creutzfeldt‐jakob disease [10]. Both classical and atypical prion strains share the defining property of transmissibility, though their molecular and pathological characteristics differ significantly. Creating models that spontaneously generate classical prions has proven particularly difficult, whereas several systems now effectively model atypical strains. Notable examples include mice expressing mutant PrP that causes GSS syndrome in humans (P102L and A117V variants) [11, 12], bank vole PrP transgenic models [4], and transgenic mice replicating key features of Nor98 atypical scrapie [6]. These systems share several common elements: they produce PrP conformers with unusual fragmentation patterns after protease digestion, they show a dose‐dependent relationship between expression level and disease onset, and the resultant prions typically propagate efficiently within the same model but, with some exceptions [6], poorly in WT animals. A key feature across these models is the requirement for either a pathogenic mutation or a specific polymorphic variant of PrP that enhances misfolding propensity. The pioneering work of Watts and colleagues with bank vole PrP established a crucial model system [4]. Bank voles are remarkably susceptible to prions from various species, but Watts' research highlighted the specific role of the I109 polymorphism in promoting spontaneous disease. Position 109 in bank vole PrP (corresponding to position 108 in mouse PrP) appears to be a critical determinant of protein stability and misfolding propensity. Although isoleucine at this position is not unique to bank voles [13, 14]—appearing naturally in at least six other mammalian species—it does not cause apparent spontaneous disease in those other species. Building on these findings, among the most significant recent developments was the work by Vidal et al., who created mice expressing sheep PrP carrying isoleucine at position 112 [6]. This single amino acid substitution, which occurs naturally in Tibetan sheep, resulted in spontaneous neurological disease with molecular and pathological features identical to Nor98 atypical scrapie. Together with bank vole studies, this work demonstrated that a single naturally occurring polymorphism, when overexpressed, can drive spontaneous formation of infectious atypical prions that precisely match those found in natural disease. Position 108 has long held significance in mouse prion biology, as the classical *Prnp*(a) and *Prnp*(b) genotypes differ at this position (leucine vs. phenylalanine, respectively). This natural variation influences strain propagation characteristics and has been used for strain typing for decades [2, 15, 16]. The differential susceptibility patterns observed between these genotypes established position 108 as a critical determinant of prion‐host interactions, with *Prnp*(a) mice (L108) showing greater susceptibility to various scrapie isolates compared to *Prnp*(b) mice (F108) [2, 15, 16]. Recent in vitro experiments by Pérez‐Castro et al. have provided molecular insights into this position's importance. Through systematic replacement of the native leucine at position 108 in mouse PrP with each of the 20 standard amino acids, they demonstrated that isoleucine uniquely accelerates spontaneous misfolding compared to all other substitutions [17]. This finding offers a biochemical explanation for the enhanced misfolding propensity observed in transgenic models expressing I108 variants and suggests specific structural consequences of this substitution that facilitate the transition from PrP^C^ to pathological conformers.

Here, we systematically examine how the L108I substitution affects prion biology in mice under different expression conditions. By creating both an overexpression transgenic line and a CRISPR‐Cas generated knock‐in model with physiological expression levels, we dissect the relative contributions of amino acid identity and expression level to spontaneous prion formation. Our results demonstrate that L108I substitution alone is insufficient to cause spontaneous disease without overexpression, highlighting the dual requirements for both sequence susceptibility and elevated protein levels. The L108I overexpression model exhibits exceptional versatility in propagating diverse prion strains, including both classical and atypical conformers, and shows strain‐specific transmission barriers. These properties make it an ideal system for investigating strain adaptation, cross‐species transmissibility, and potential therapeutic approaches targeting prion diseases.

## 2 MATERIALS AND METHODS

### 2.1 Ethics statement—animal welfare approvals and regulatory compliance

This study was conducted following strict adherence to European Union directives and Spanish national legislation regarding the protection of animals used for scientific purposes. All procedures received prior authorization from the corresponding institutional animal welfare ethics committees.

#### 2.1.1 Transgenic mouse generation

Both TgMo(L108I)3x and TgMo(L108I)1x mouse lines were developed at the Transgenic Facility of the IBSAL, University of Salamanca, Spain, under ethical approval code JCyL‐1084 from the Institutional Animal Welfare Ethics Committee.

#### 2.1.2 Multi‐institutional experimental procedures

Animal studies were carried out across several Spanish research centers, including CIC bioGUNE, University of Santiago de Compostela (Centre for Experimental Biomedicine [CEBEGA]), Neiker—Basque Institute for Agricultural Research and Development, and IRTA‐CReSA Animal Health Research Center. Each institution obtained independent ethical approval through their respective animal welfare committees under the following authorization codes: CIC bioGUNE (P‐CBG‐CBBA‐0314 and 15005/16/006), CEBEGA (15012/2023/002), and Neiker (NEIKER‐OEBA‐2021‐003).

#### 2.1.3 Regulatory framework

Experimental procedures conducted prior to 2013 were performed under the regulatory framework established by “Real Decreto 1201/2005 de 10 de Octubre” and “Real Decreto 214/1997 de 30 de Julio” governing animal welfare in experimental research. Subsequent studies (2013 onwards) were conducted in compliance with “Real Decreto 53/2013 de 1 de febrero” on the protection of laboratory animals, which implements European Directive 2010/63/EU on Laboratory Animal Protection into Spanish national law.

### 2.2 Generation and characterization of transgenic mice

#### 2.2.1 Development of TgMo(L108I)3x mice via conventional transgenesis

##### Vector construction and molecular cloning

The mouse PrP (MoPrP) open reading frame (ORF) originally encoding leucine at codon 108 (GenBank accession number NM_011170) was obtained and subjected to site‐directed mutagenesis using standard molecular biology techniques to introduce the leucine‐to‐isoleucine substitution at position 108. The modified sequence was subsequently cloned into the MoPrP.Xho vector [18], which harbors the murine *Prnp* regulatory elements, including the promoter, exon‐1, intron‐1, exon‐2, and 3′ untranslated regions that ensure neuronal‐specific transgene expression. Prior to microinjection procedures, the transgenic construct was released from the vector backbone through *Not*I digestion (New England Biolabs, USA) followed by purification. The microinjection‐ready DNA fragment was isolated using the Qiagen gel extraction system (Qiagen, Germany) and resuspended in TE buffer (10 mM Tris, 0.25 mM ethylenediamine tetraacetic acid [EDTA], pH 7.5) at a final concentration of 5 ng/μL.

##### Pronuclear microinjection and founder screening

Transgenic founder mice were established through pronuclear microinjection of the purified transgenic DNA into fertilized oocytes derived from C57BL/6xCBA F1 matings using established protocols [19]. Following microinjection of 138 embryos and transfer to pseudopregnant recipients, 18 live‐born animals were obtained, three of which developed into independent transgenic founder lines. While one founder line failed to produce viable offspring, the remaining two successfully transmitted the transgene through the germline. Transgenic founders were identified through polymerase chain reaction (PCR) screening of tail biopsy‐derived DNA using primer pairs specific for mouse exon‐2 and 3′ untranslated regions (5′‐GAACTGAACCATTTCAACCGAG‐3′ and 5′‐AGAGCTACAGGTGGATAACC‐3′). PCR‐positive founder animals were subsequently crossed with *Prnp*‐knockout mice (129/Ola‐*Prnp* ^0/0^ strain) [20] to eliminate endogenous mouse prion protein expression. Successful elimination of the endogenous *Prnp* allele was verified by PCR amplification using primers 5′‐ATGGCGAACCTTGGCTACTGGC‐3′ and 5′‐GATTATGGGTACCCCCTCCTTGG‐3′.

#### 2.2.2 Development of TgMo(L108I)1x knock‐in mice via CRISPR‐Cas9 genome editing

##### Guide RNA design and CRISPR components preparation

For the development of the *Prnp* (c.322 C>A p.108L>I) knock‐in mouse model, we employed the CRISPR‐Cas9 system with chemically synthesized components (Integrated DNA Technologies), including tracrRNA, one specific crRNA targeting exon 1, and a designed 200‐nucleotide single‐stranded DNA template (see Figure S1). Mature single‐guide RNA (sgRNA) was obtained by annealing equimolar amounts of crRNA and tracrRNA through a heating and cooling cycle.

##### Microinjection and founder generation

A solution containing the mature sgRNA (20 ng/μL), recombinant Cas9 protein (30 ng/μL), and the single‐stranded DNA (ssDNA) (10 ng/μL) were microinjected into the pronucleus of B6J/CBA mouse zygotes at the Transgenic Facility of the IBSAL‐University of Salamanca. Edited founders were identified by PCR amplification (Taq polymerase, NZYtech) with primers flanking the edited region (see Figure S1).

##### Validation and colony establishment

PCR products were analyzed by direct sequencing or through subcloning into pBlueScript vectors (Stratagene) followed by Sanger sequencing. Selected founders carrying the desired allele were backcrossed with B6J/CBA WT animals to eliminate potential off‐target mutations. Heterozygous offspring were re‐sequenced and subsequently crossed with B6J/CBA WT animals to establish the *B6J/CBA‐PrnP1* ^*em1(c322C>A)Msam*^ colony, referred to throughout this manuscript as TgMo(L108I)1x.

#### 2.2.3 Common methodological procedures

##### DNA extraction and quality assessment

Genomic DNA was isolated from tail biopsies using the QIAamp® DNA Mini Kit (QIAGEN, Germany) according to the manufacturer's tissue purification protocol. DNA quantification and purity evaluation were performed using ultraviolet spectrophotometry on a NanoVue Plus spectrophotometer (GE Healthcare, USA), measuring absorbance at 260, 280, and 230 nm wavelengths. Sample integrity was assessed through electrophoretic separation on 1.5% agarose gels prepared with 1× Tris‐acetate‐EDTA buffer (Fisher Scientific, USA) and visualized under ultraviolet (UV) transillumination.

##### Amplification of a Prnp fragment and sequence verification

To confirm the leucine‐to‐isoleucine substitution at residue 108, a specific 339‐bp fragment of the murine *Prnp* gene was amplified using primers designed with FastPCR (v.3.3.67) and validated through National Center for Biotechnology Information Primer‐BLAST for optimal thermodynamic properties and target specificity. Amplification primers incorporated M13 sequencing adaptors (shown in italics) for streamlined sequencing workflows (FM13_PRNP: 5′‐*TGTAAAACGACGGCCAGT*CCATAATCAGTGGAACAAGC‐3′; RM13_PRNP: 5′‐*CAGGAAACAGCTATGACC*CGCTCCATCATCTTCACATC‐3′). PCR reactions were performed in 10 μL volumes containing 5.75 μL nuclease‐free water, 1.0 μL 10× NH_4_ reaction buffer, 0.4 μL MgCl_2_ (50 mM stock), 0.25 μL deoxyribonucleoside triphosphate mixture (100 mM stock), 1.5 μL primer cocktail (2.5 μM each), 0.1 μL BIOTAQ™ DNA polymerase (Bioline GmbH; 5 U/μL), and 2.0 μL genomic DNA template. Optimal annealing temperature was determined through gradient PCR (50–65°C) using pooled DNA samples, with 65°C selected for routine amplifications. PCR products underwent enzymatic cleanup using ExoSAP‐IT treatment (37°C for 15 min, followed by 80°C enzyme inactivation for 15 min) before Sanger sequencing analysis using BigDye Terminator v3.1 chemistry on an ABI 3500 Genetic Analyzer (Fisher Scientific).

##### Transgene expression characterization

L108I MoPrP expression levels in brain tissue from transgenic offspring lacking endogenous mouse PrP were quantified through Western blot analysis using anti‐PrP^C^ monoclonal antibody SHa‐31 (1:4000 dilution) with WT mouse brain homogenates serving as reference standards. The selected line, designated TgMo(L108I)3x, demonstrated L108I mouse PrP expression at approximately three‐fold WT levels in heterozygous animals, maintaining normal glycosylation patterns as confirmed by Western blot analysis (Figure S2). This line was chosen for experimental studies and maintained in a hemizygous state through continuous backcrossing to 129/Ola‐*Prnp* ^0/0^ mice. The formal nomenclature for this transgenic line is B6&CBA.129Ola‐Tg(Prnp‐Mo108I)1Sala/Cicb, referred to as TgMo(L108I)3x throughout this manuscript. Similarly, the CRISPR‐Cas9 generated knock‐in line expressing L108I mouse PrP at physiological levels carries the official designation B6&CBA‐*Prnp* ^*tm1Mo108ISala*^/Cicb and is referred to as TgMo(L108I)1x throughout this study.

### 2.3 Preparation of brain homogenates and inocula

Brain tissue specimens were harvested immediately following humane euthanasia from both clinically affected and asymptomatic animals. For characterization of spontaneous disease, mice were euthanized via carbon dioxide asphyxiation upon manifestation of neurological symptoms, following institutional animal welfare protocols. Following extraction, brains were bisected sagittally with one hemisphere preserved at −80°C for biochemical evaluation and the contralateral hemisphere immersion‐fixed in 10% neutral buffered formalin (Sigma‐Aldrich, USA) for histopathological examination.

Brain homogenate preparation involved thawing frozen tissue samples followed by mechanical homogenization to achieve 10% (w/v) suspensions in phosphate‐buffered saline (PBS, Fisher Bioreagents) supplemented with Complete Protease inhibitor mixture (Roche) using glass Potter‐Elvehjem homogenizers (Fisher Scientific). Homogenized samples were portioned into aliquots and maintained at −80°C until subsequent analysis or experimental use. For stereotaxic inoculation procedures, the 10% homogenates underwent serial dilution to 1% concentrations in Dulbecco's PBS (DPBS, Gibco).

### 2.4 Prion transmission experiments

#### 2.4.1 Intracerebral inoculation

Mice aged 6–8 weeks underwent anesthesia using either isoflurane inhalation (IsoVet, Braun) or ketamine/medetomidine combination therapy (75/1 mg/kg) (Imalgene 1000, Boehringer Ingelheim/Domtor, Ecuphar). When employing the latter protocol, anesthetic reversal was achieved using atipamezole hydrochloride (1 mg/kg) (Antisedan, Ecuphar). A minute cranial opening was established in the right parietal region, allowing delivery of 20 μL of 1% brain homogenate into the right cerebral parenchyma at approximately 3 mm depth utilizing a precision microsyringe equipped with a sterile 27‐gauge needle (Terumo). To minimize inoculum backflow through the injection channel, the needle was maintained in position for 20 s prior to controlled withdrawal.

#### 2.4.2 Post‐inoculation clinical surveillance

Subsequent to inoculation, experimental animals were group‐housed (5–6 mice per enclosure) under standardized conditions (ambient temperature 22°C, alternating 12‐h photoperiods, 60% relative humidity) within HEPA‐filtered, independently ventilated housing systems. Animals maintained unrestricted access to standard laboratory diet and underwent daily welfare assessments. Comprehensive clinical evaluations were conducted bi‐weekly until initial neurological manifestations emerged, at which point surveillance frequency increased to daily examinations.

Clinical manifestations consistent with prion pathology were quantified using a graduated scoring system (0–3) encompassing the following criteria: spinal curvature abnormalities, locomotor dysfunction, coat quality deterioration, behavioral depression, postural changes, ocular discharge, behavioral hyperexcitability, body condition decline, and eliminatory incontinence. Animals demonstrating persistent clinical abnormalities (scores ≥2 across multiple parameters) or severe neurological compromise affecting welfare underwent humane termination. Incubation intervals were quantified as the duration from inoculation to euthanasia, reported as days post‐inoculation (dpi).

Transmission efficiency was calculated as the proportion of animals developing laboratory‐confirmed prion disease (through histopathological and/or biochemical validation) relative to the total inoculated cohort. Animals succumbing to intercurrent illness or exhibiting non‐specific pathology prior to reaching 50% of the group's average incubation duration were excluded from analysis. Data presentation follows the format of mean incubation duration ± standard error of the mean (SEM) for individual experimental cohorts.

### 2.5 Biochemical analysis of misfolded prion protein

Brain specimens from both spontaneously affected and experimentally inoculated mice underwent biochemical evaluation to identify and characterize pathological prion protein conformers. Frozen brain tissue samples were thawed and mechanically disrupted to achieve 10% (w/v) suspensions in PBS (Fisher Bioreagents) supplemented with Complete Protease Inhibitor Cocktail (Roche) utilizing glass Potter homogenizers (Fisher Scientific). For comparative biochemical validation, brain homogenates derived from classical scrapie isolate SSBP/1 (TSE Resource Center, University of Edinburgh) and atypical scrapie strain Nor98 (generously provided by Olivier Andréoletti, INRAE) served as reference standards.

#### 2.5.1 Detection of classical PrP^res^

Classical PrP^res^ exhibiting the typical tripartite banding pattern was detected through conventional PK proteolysis. Sample preparation involved combining homogenates 1:1 (v/v) with enzymatic digestion medium [comprising 2% (w/v) Tween‐20 (Sigma‐Aldrich), 2% (v/v) NP‐40 (Sigma‐Aldrich), and 5% (w/v) Sarkosyl (Sigma‐Aldrich) dissolved in PBS] achieving 5% final brain homogenate concentrations. PK (Roche) was incorporated at final concentrations ranging from 85 to 170 μg/mL, followed by incubation at 42°C for 1 h under moderate agitation (450 rpm). Enzymatic digestion was terminated through the addition of NuPAGE 4x loading buffer (Invitrogen, USA) at 1:3 (v/v) ratios and thermal denaturation at 100°C for 10 min. This standard proteolysis approach was consistently employed for classical prion strain analysis, encompassing SSBP/1, Rocky Mountains Laboratory mouse prion strain (RML), 22L, 301C, TgSh112I [6], and laboratory‐adapted CWD‐TgVole isolates.

#### 2.5.2 Detection of atypical PrP^res^

Atypical PrP^res^ displaying characteristic ladder‐like electrophoretic patterns and prominent low molecular weight fragments (7–10 kDa) was detected using a specialized protocol adapted from Wenborn et al. [21]. Initial processing involved treating brain homogenates (10% w/v in PBS) with Pronase E (Sigma‐Aldrich) at 100 μg/mL concentrations for 30 min at 37°C under vigorous agitation (800 rpm). Subsequently, EDTA (Calbiochem) and Sarkosyl were added to achieve final concentrations of 10 mM and 2% (w/v), respectively, before secondary enzymatic treatment with Benzonase (Merck) at 50 U/mL for 10 min at 37°C with continued mixing. Sodium phosphotungstic acid (NaPTA, Sigma‐Aldrich) was incorporated to 0.3% (w/v) final concentration, and samples underwent 30‐min incubation at 37°C. Following density gradient preparation by combining samples with 60% iodixanol solution (OptiPrep density gradient medium, Sigma‐Aldrich) to achieve 35% (v/v) iodixanol and 0.3% (w/v) NaPTA final concentrations, samples were subjected to high‐speed centrifugation at 16,100 *g* for 90 min. Resulting supernatants were combined 1:1 with buffering solution containing 2% Sarkosyl (w/v) and 0.3% NaPTA in PBS, followed by secondary 90‐min centrifugation at 16,100 g. After supernatant removal, pellets were reconstituted in washing solution (17.5% [w/v] iodixanol and 0.1% [w/v] Sarkosyl in PBS). Reconstituted pellet fractions underwent PK proteolysis at 10 μg/mL final concentrations for 1 h at 37°C. Following washing buffer addition and NaPTA incorporation to 0.3% (w/v) final concentration, samples were centrifuged for 30 min at 16,100 *g* with supernatant disposal. This purification cycle was repeated once, and final pellets were resuspended in NuPAGE 4x loading buffer (Invitrogen, USA) diluted 1:3 (v/v) with PBS. This specialized methodology proved essential for detecting atypical prion conformers from spontaneously affected TgMo(L108I)3x mice, homologous secondary transmissions, TgMo(L108I)3x animals exposed to heterologous atypical prions, and corresponding source isolates including Nor98 atypical scrapie, TgSh112I, and A117V GSS specimens [22].

#### 2.5.3 Western blot analysis

Post‐digestion samples underwent thermal denaturation at 100°C for 10 min before electrophoretic separation on 4%–12% Bis‐Tris polyacrylamide gel systems (NuPAGE, Invitrogen). Electrophoresis proceeded at 200 V for approximately 80 min. Protein transfer to polyvinylidene difluoride membranes (Trans‐Blot Turbo Transfer Pack, Bio‐Rad, USA) utilized the Trans‐Blot Turbo Transfer System (Bio‐Rad). Membrane blocking employed 5% non‐fat milk in Tris‐buffered saline+Tween‐20 (TBST) buffer (TBS supplemented with 0.1% Tween‐20) for 1 h at ambient temperature, followed by overnight primary antibody incubation at 4°C. Mouse PrP detection utilized monoclonal antibody 9A2 (1:2000 dilution, recognizing WNK epitope). Following triple TBST washing, membranes were exposed to horseradish peroxidase‐conjugated secondary antibodies (anti‐mouse IgG or anti‐human IgG, 1:5000 dilution, Santa Cruz Biotechnology) for 1 h at room temperature. After additional washing cycles, immunoreactive signals were developed using enhanced chemiluminescent substrate (West Pico Plus, Thermo Scientific) and captured using FluorChem Q (Alpha Innotech) or iBright CL750 (Invitrogen) imaging platforms. Quantitative analysis and densitometry were conducted using AlphaView software (Alpha Innotech, USA) or ImageJ (NIH, USA).

### 2.6 Immunohistochemistry

#### 2.6.1 Tissue preparation and processing

Brain specimens were harvested and immersion‐fixed in 10% neutral buffered formalin (Sigma‐Aldrich) for 24–48 h at ambient temperature. Post‐fixation, brain tissues underwent transverse sectioning at three anatomical levels: medulla oblongata (Mob), piriform cortex, and optic chiasm regions. Tissue samples were subsequently dehydrated through graduated ethanol concentrations (70%, 96%, and 100%) followed by xylene clearing prior to paraffin wax infiltration and embedding. Serial sections of 4‐μm thickness were generated using rotary microtomy and transferred onto standard glass microscopy slides for hematoxylin and eosin (H&E) histological evaluation of spongiform pathology, neuronal depletion, and glial proliferation. For immunohistochemical applications, parallel sections were mounted on 3‐triethoxysilyl‐propylamine‐treated glass slides (DAKO, Denmark) to optimize tissue retention during processing.

#### 2.6.2 PrP^res^ immunodetection

Disease‐associated prion protein visualization required deparaffinized tissue sections to undergo sequential epitope unmasking procedures. Initial treatment involved 98% formic acid immersion for 15 min, followed by thorough distilled water rinsing. Sections then underwent pressure cooker autoclaving in citrate buffer (pH 6.15) at 121°C for 20 min. Following cooling, PK digestion (4 μg/mL, Roche) was performed for 15 min at 37°C to unmask epitopes and enhance antibody accessibility. Endogenous peroxidase quenching utilized 3% hydrogen peroxide in methanol for 30 min at room temperature. Non‐specific binding sites were saturated using 10% normal goat serum in PBS supplemented with 0.1% Triton X‐100 for 30 min.

#### 2.6.3 Antibody incubation and visualization

Primary antibody detection employed anti‐PrP mouse monoclonal antibody 2G11 (Bio‐Rad) (1:100) for mouse PrP recognition in TgMo(L108I)3x/1x experimental models. Overnight primary antibody incubation occurred at 4°C within humidified chambers, followed by comprehensive PBS washing and secondary incubation with peroxidase‐conjugated polymer systems containing anti‐mouse antibodies (EnVision, DAKO) for 30 min at room temperature. Immunoreactive signals were developed using 3,3′‐diaminobenzidine (DAB, Dako) chromogenic substrate and hydrogen peroxide. Sections received hematoxylin counterstaining, ethanol dehydration, xylene clarification, and distyrene, plasticizer, and xylene mounting medium application (Sigma‐Aldrich). Negative controls involved primary antibody omission for each staining batch. Astrocytic activation was assessed through overnight incubation with rabbit polyclonal anti‐glial fibrillary acidic protein ([GFAP], 1:500, DAKO) antibodies, while microglial responses utilized goat polyclonal anti‐ionized calcium binding adapter molecule 1 (IBA1, 1:1000, Abcam, UK) antibodies. Both required heat‐mediated epitope retrieval at pH 6 (Target retrieval solution, DAKO) at 96–98°C for 20 min. GFAP detection utilized polymer‐conjugated anti‐rabbit secondary antibodies with peroxidase (EnVision, DAKO), while IBA1 employed anti‐goat polymer systems with peroxidase (ImmPRESS HRP Horse anti‐goat, Vector Laboratories, USA), both incubated for 30 min at room temperature, with DAB development as described for PrP^res^.

#### 2.6.4 Neuropathological evaluation

Spongiform degeneration and PrP^res^ immunoreactivity underwent semi‐quantitative assessment by an experienced neuropathologist under blinded experimental conditions. Fourteen neuroanatomical regions received evaluation: piriform cortex (Pfc), hippocampus (H), occipital cortex (Oc), temporal cortex (Tc), parietal cortex (Pc), frontal cortex (Fc), striatum (S), thalamus (T), hypothalamus (HT), mesencephalon (M), Mob, cerebellar nuclei (Cm), cerebellar vermis (Cv), and cerebellar cortex (Cc). The grading scheme utilized a 0–4 scale for both spongiform alterations and PrP^res^ accumulation: 0 indicating absence of pathology or immunoreactivity, 1 representing minimal changes (occasional vacuoles or scattered immunolabeling), 2 denoting moderate alterations (multiple vacuoles or moderate immunoreactivity), 3 signifying pronounced changes (extensive vacuolation or abundant immunolabeling), and 4 indicating severe pathology (confluent vacuolation or intense diffuse immunoreactivity). Lesion distribution profiles were constructed by plotting average scores across brain regions arranged along the caudo‐rostral neuraxis.

### 2.7 Statistics and reproducibility

#### 2.7.1 Experimental design and sample sizing

Animal group sizes were established based on prior experience with comparable transgenic prion models and standard practices in prion bioassay protocols. Spontaneous disease monitoring utilized 85 animals observed over a 10‐year period to provide robust survival analysis data. Transmission bioassays employed groups of five to nine animals per experimental condition, consistent with established prion transmission protocols.

#### 2.7.2 Data analysis and statistical methods

Statistical evaluations were conducted using GraphPad Prism (version 9.0, GraphPad Software Inc.) and R software (version 4.3.0). Data presentation follows the format of mean ± SEM unless specified otherwise. Survival data underwent Kaplan–Meier analysis with log‐rank testing for inter‐group comparisons. Sex‐based comparisons of disease onset employed Student's *t*‐tests following confirmation of data normality through Shapiro–Wilk testing. Multi‐group analyses utilized analysis of variance (ANOVA) with appropriate post hoc corrections.

#### 2.7.3 Statistical significance criteria

The threshold for statistical significance was established at *p* < 0.05 for all comparisons. Where applicable, corrections for multiple testing were implemented to maintain statistical rigor. Experiments incorporated suitable control groups and blinded assessment procedures when operationally feasible.

## 3 RESULTS

### 3.1 Generation of transgenic mouse models expressing L108I PrP: Overexpression and knock‐in strategies

To investigate the effect of the L108I substitution on prion biology under different expression conditions, we developed two distinct transgenic mouse models using complementary genetic approaches. For the first model, we engineered mice overexpressing mouse PrP with an isoleucine substitution at position 108. The ORF of mouse PrP carrying the L108I mutation was inserted into the pJB vector containing the mouse PrP promoter and regulatory sequences that direct transgene expression primarily to neuronal tissues. After linearization and purification, the construct was microinjected into fertilized C57BL6xCBA embryos following standard procedures. One‐hundred and thirty‐eight embryos were microinjected, resulting in 18 animals born. Five founder (F0) animals were obtained that successfully transmitted the transgene to their offspring, which were subsequently backcrossed to 129/Ola‐Prnp^0/0^ mice [20] to eliminate endogenous mouse PrP expression and maintained in hemizygous state. Founder animals were identified by PCR analysis of tail biopsy DNA using specific primers targeting the transgene construct. Among the various transgenic lines generated, we selected the line with the highest expression level, designated TgMo(L108I)3x, which expressed the mutant PrP at approximately three times the level found in WT mouse brain when in heterozygous state. Western blot analysis confirmed that the glycosylation pattern remained unaltered compared to WT PrP (Figure S2). Integration analysis suggested X‐chromosome insertion, as evidenced by complete male sterility in this line, which required exclusive breeding through females. For the second model, we employed CRISPR‐Cas9 technology to create a precise knock‐in of the L108I mutation while maintaining physiological expression levels. Guide RNA was designed to target the specific region of the *Prnp* gene containing codon 108. The repair ssDNA template incorporated the single nucleotide change required to convert the leucine codon to isoleucine, while preserving all regulatory elements of the endogenous gene. Microinjections were performed in C57BL/6 embryos, resulting in five viable founder animals (F0). Amplicon sequencing of the PrP coding region confirmed the precise integration of the desired mutation without additional alterations. Selected founder animals were crossed with WT C57BL/6 mice, and heterozygous offspring were intercrossed to establish homozygous knock‐in lines. Careful analysis of PrP expression levels in homozygous animals confirmed that the L108I variant was expressed at levels equivalent to WT PrP in non‐transgenic mice (Figure S2).

### 3.2 Spontaneous neurological disease development in TgMo(L108I)3x mice

All TgMo(L108I)3x mice developed a spontaneous neurological disease characterized by ataxia, circling, dysmetria, kyphosis, and proprioceptive deficits. Animals reached terminal disease between 219 and 536 days of age, typically progressing through a relatively consistent clinical phase lasting 10–15 days after initial detection of signs, ultimately leading to end‐stage disease. Animals were humanely euthanized upon reaching predefined endpoints according to standardized clinical scoring criteria, following institutional animal care guidelines. To comprehensively assess the temporal progression of disease development, we evaluated a total of 85 mice observed over the period 2014–2024 and investigated potential sex‐dependent differences in terminal disease timing, given previous observations in TgVole(I109)4x mice [7].

**FIGURE 1:**
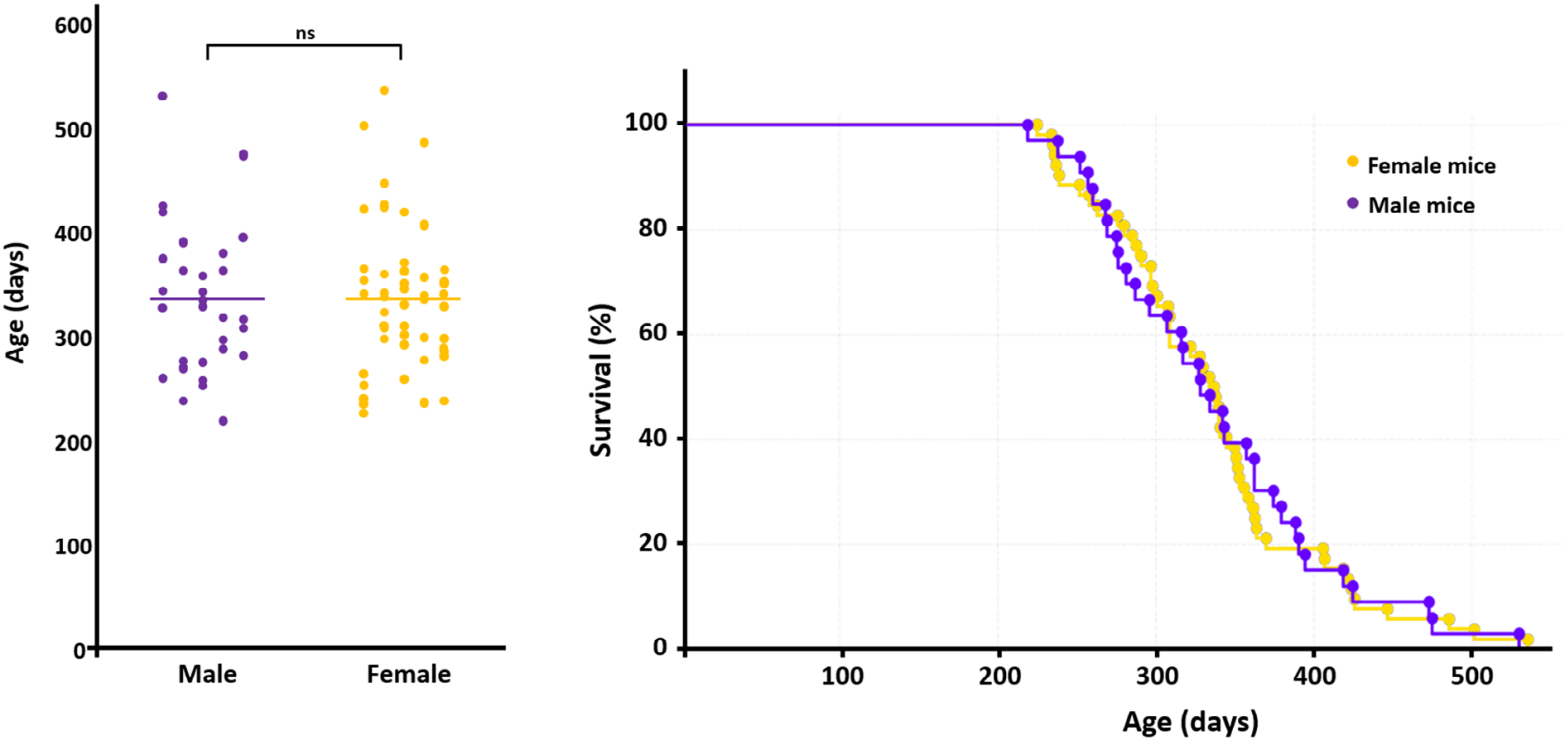
Terminal disease timing in male and female TgMo(L108I)3x mice with spontaneous prion disease. Neurological signs were monitored in 85 TgMo(L108I)3x mice over 10 years to determine the range and mean age at which animals reached terminal disease endpoints. Terminal disease timing was analyzed separately by sex given prior reports of sex‐dependent differences in the TgVole(I109)4x spontaneous prion disease model [7]. Male mice had a mean terminal disease age of 337 ± 12.7 days, similar to females at 335 ± 9.5 days (Student's *t*‐test, *p* = 0.423, not significant [ns]). Kaplan–Meier survival curves (right plot) confirm nearly identical terminal disease distributions: 80% of males reached endpoints between 257 and 418 days and females between 252 and 370 days, with similar individual variability.

Both sexes exhibited similar disease progression patterns, with no statistically significant sex‐dependent differences in terminal disease timing. Male mice displayed a mean terminal disease age of 337 ± 12.7 days, while females showed a comparable mean at 335 ± 9.5 days. Statistical analysis using Student's *t*‐test confirmed the lack of significant differences between sexes (*p* > 0.05) (Figure 1). The neurological manifestations were identical in both males and females, with all affected animals exhibiting the same characteristic disease syndrome, with prominent paralysis of the hind limbs. Analysis of terminal disease distribution revealed that 80% of male mice reached endpoints between 257 and 418 days of age, while 80% of females showed terminal disease between 252 and 370 days of age.

In contrast, age‐matched TgMo(L108I)1x mice monitored beyond 600 days of age failed to develop any neurological abnormalities, demonstrating that the expression level of the transgene plays a critical role in the spontaneous development of prion disease in this model.

### 3.3 Pathological and biochemical characterization of spontaneous prion disease in TgMo(L108I)3x mice

**FIGURE 2:**
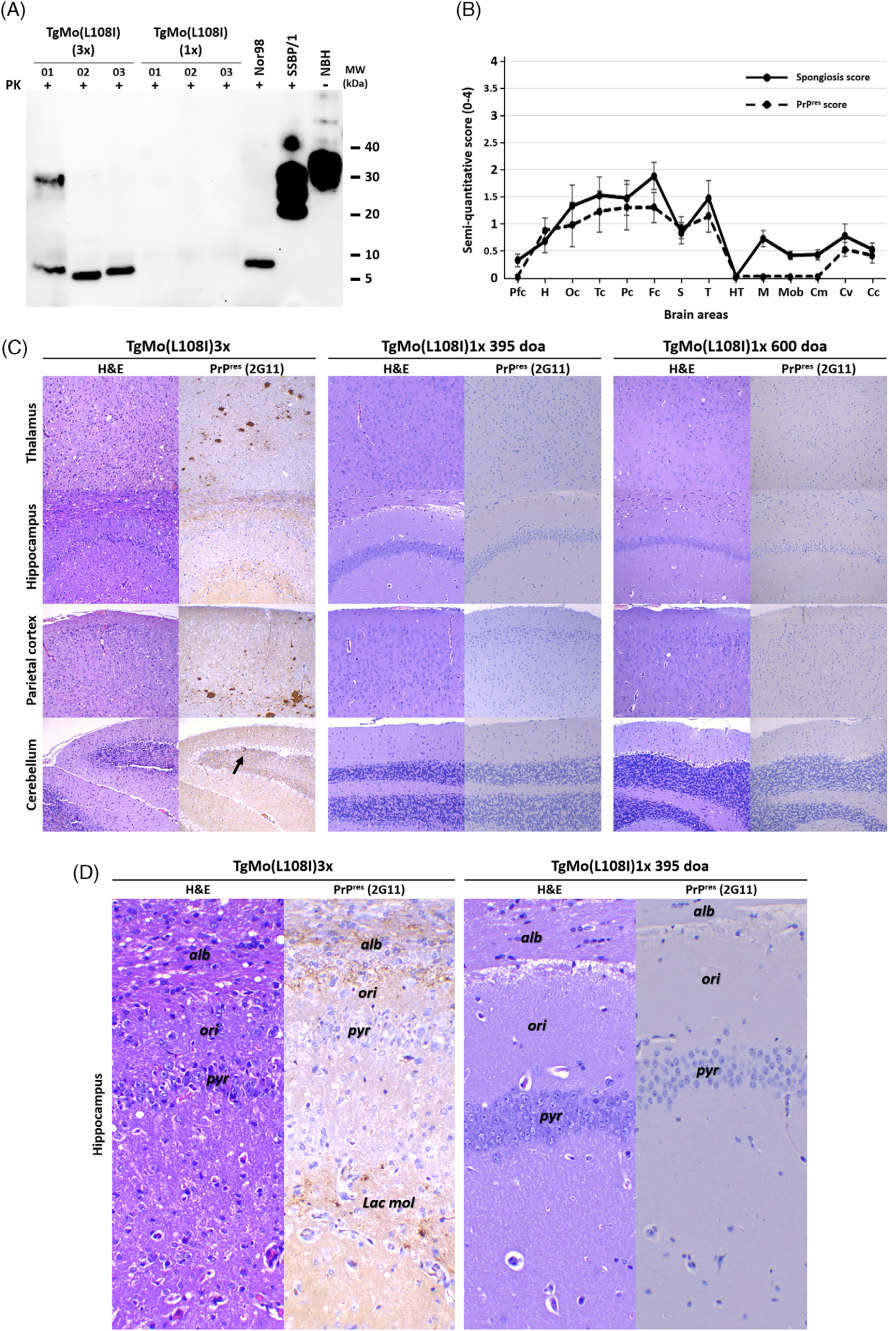
Biochemical and histopathological characterization of the spontaneous prion disease in TgMo(L108I)3x mice compared to aged TgMo(L108I)1x mice. (A) Biochemical characterization of proteinase‐resistant PrP in brains from spontaneously ill TgMo(L108I)3x mice and aged healthy TgMo(L108I)1x mice. Western blot analysis of proteinase K (PK)‐resistant PrP from 10% (w/v) brain homogenates of TgMo(L108I)3x mice (three representative animals culled at 302, 338, and 350 days of age with clear signs of disease) and from aged TgMo(L108I)1x mice (culled at >600 days of age) was performed, after processing all samples using the modified Wenborn protocol for atypical prion detection [21]. All samples were digested with 10 μg/mL PK and revealed a prominent low molecular weight fragment (~7 to 10 kDa) in all spontaneously ill TgMo(L108I)3x animals, completely absent in TgMo(L108I)1x mice of more than 600 days of age. For comparison, atypical scrapie isolate (Nor98) processed identically, and classical scrapie isolate (SSBP/1) digested at 85 μg/mL PK and without further processing are shown as controls. An undigested brain homogenate from a healthy TgMo(L108I)3x mouse (normal brain homogenate) serves as negative control. Detection was performed using 9A2 monoclonal antibody (1:4000). MW, molecular weight marker. (B) Semi‐quantitative (0–4) spongiform lesion and PrP^res^ deposition pattern in spontaneously ill TgMo(L108I)3x mice determined by histopathological and immunohistochemical analysis. Spongiform lesion (solid black line) was most intense in neocortex and thalamus, moderate in striatum and mesencephalon and mild in the cerebellar cortex. PrP^res^ deposits (dashed line), detected using 2G11 anti‐PrP mAb, are more variable between animals with some showing only small multifocal plaques, others showing a granular pattern that coalesces into multifocal plaques and few animals with only the granular pattern, in most cases correlating well with spongiform changes. Brain regions: Pfc (piriform cortex), H (hippocampus), Oc (occipital cortex), Tc (temporal cortex), Pc (parietal cortex), Fc (frontal cortex), S (striatum), T (thalamus), HT (hypothalamus), M (mesencephalon), Mob (medulla oblongata), Cm (cerebellar nuclei), Cv (cerebellar vermis), Cc (cerebellar cortex). (C) Representative brain sections stained with hematoxylin and eosin (H&E) and immunostained for PrP^res^ detection from TgMo(L108I)3x mice and from age‐matched and aged TgMo(L108I)1x mice. Neuropathological characterization of the spontaneous pathology in spontaneously ill TgMo(L108I)3x mice (left panel) compared to healthy age‐matched (395 days of age, middle panel) and aged (600 days of age, right panel) healthy TgMo(L108I)1x mice. H&E staining is shown on the left of each panel and immunohistochemistry against PrP^res^ (2G11 mAb 1:100) on the right side. Notice intense spongiform change, increased cell numbers (gliosis) and conspicuous plaques along with granular immunolabeling pattern in the thalamus and parietal cortex of the TgMo(L108I)3x mice, in which the cerebellar cortex shows evident loss of granules in the granular cell layer with discrete immunolabeled PrP^res^ aggregates (arrow), whereas no changes or immunolabeling were observed in the TgMo(L108I)1x mice except for mild spongiosis in the 600‐day‐old group, likely associated with aging. (D) Detail of hippocampal lesions from TgMo(L108I)3x and age‐matched TgMo(L108I)1x mice. The hippocampus shows atrophy with severe loss of pyramidal cells, gliosis and fine punctate immunolabeling pattern in TgMo(L108I)3x mice. H&E staining is shown on the left and immunohistochemistry against PrP^res^ (2G11 mAb 1:100) on the right. Hippocampal layers are indicated: *Alb* (alveus), *ori* (oriens), *pyr* (pyramidal), *Lac mol* (lacunosum moleculare). No pathological changes or PrP^res^ immunolabeling were observed in age‐matched TgMo(L108I)1x mice (395 days of age). doa, day of age.

Biochemical analysis of brain homogenates from TgMo(L108I)3x mice exhibiting clinical signs revealed an atypical prion protein pattern following proteinase K digestion. All affected animals demonstrated a characteristic low molecular weight band of approximately 7–10 kDa (Figure 2A), remarkably similar to the signature observed in other atypical prion disorders such as Nor98 in sheep. This biochemical profile differs substantially from the three‐band pattern typically associated with classical prion diseases, suggesting a unique conformational state of the misfolded prion protein.

Histopathological and immunohistochemical examination revealed a consistent phenotype (Figure 2B,C). We observed intense spongiform change in the neocortex and thalamus, with no involvement of the HT, moderate spongiosis in the striatum and mesencephalon, and mild changes in the Mob and Cc. The Cc showed evident loss of granule cells from the granular cell layer. Some variability was observed among animals, likely because of differences in disease progression timing upon euthansia, as illustrated by the conspicuous loss of pyramidal cells in the hippocampal *Cornu Ammonis* observed only in some animals (3/10) (see details in Figure 2D).

Immunohistochemistry with the anti‐PrP 2G11 mAb showed intense basal PrP^C^ labeling, consistent with observations in other overexpressing models, and displayed a variable PrP^res^ pattern, again likely because of differences in disease stage. While some animals showed only small multifocal PrP^res^ plaques, others presented with a granular pattern that occasionally coalesced into multifocal plaques, which in some animals were very abundant. A few animals showed only the granular pattern with little or no plaques, which presumably would evolve over time to form plaques (Figure 2B–D). The plaques were often associated to (or rather infiltrated with) with multiple nuclei, likely glial. The distribution of such deposits correlated well with that of the spongiform changes.

Neuroinflammatory response was a prominent feature of the spontaneous pathology in TgMo(L108I)3x mice, characterized by intense astrogliosis and microgliosis (Figure S3). GFAP immunohistochemistry revealed marked astrocytic activation with both hyperplasia and hypertrophy, particularly prominent in the neocortex (especially parietal cortex), hippocampus, thalamus, and Cc. Similarly, IBA1 immunostaining demonstrated robust microglial activation following the same anatomical distribution as the spongiform lesions and PrP^res^ deposits. Notably, in the hippocampus, microgliosis was restricted to areas with PrP^res^ deposits, suggesting a direct relationship between protein aggregation and inflammatory response. Interestingly, while PrP^res^ plaques were heavily infiltrated with activated microglia, astrocytic infiltration of plaques was not observed. The striatum, mesencephalon, and Mob showed less pronounced glial activation, paralleling the distribution pattern observed for spongiform changes and PrP^res^ deposition.

In striking contrast, age‐matched TgMo(L108I)1x mice (395 days of age) and mice from the same line monitored beyond 600 days of age showed no detectable PrP^res^ deposits, neuroinflammation, or any other neuropathological alterations associated with prion disease (Figures 2C and S3). Additionally, biochemical analysis revealed no evidence of the characteristic low molecular weight band (7–10 kDa) that serves as the hallmark of atypical prion disease in these mice (Figure 2A). This observation further confirms that transgene expression level is a critical determinant for spontaneous prion formation in this model.

### 3.4 RT‐QuIC analysis confirmed the presence of seeding‐competent prions exclusively in TgMo(L108I)3x mice

To further validate the prion nature of the spontaneous disease, brain homogenates from age‐matched animals (~350 days) from all models were subjected to real time‐quacking induced conversion (RT‐QuIC) analysis using recombinant bank vole I109 PrP as substrate, which has demonstrated superior sensitivity for detecting atypical prion strains. Brain extracts from three TgMo(L108I)3x mice showing clinical signs demonstrated robust seeding activity at both 10^−3^ and 10^−4^ dilutions, comparable to the Nor98 atypical scrapie positive control. In contrast, no seeding activity was detected in brain extracts from TgMo(L108I)1x, Tga20, or WT C57BL/6 mice at either dilution tested (Figure S4). These results confirm that seeding‐competent prions are generated exclusively in the overexpression model, supporting the dual requirement for both the L108I substitution and elevated PrP expression levels for spontaneous prion formation. Furthermore, in agreement with the RT‐QuIC results, a comparative biochemical analysis performed with 400 days old Tga20 mice and age‐matched TgMo(L108I)3x mice (334–357 days old) demonstrated that PK‐resistant prions are found only in the TgMo(L108I)3x model and thus are not a consequence of PrP overexpression alone (Figure S5).

### 3.5 Spontaneously generated prions from TgMo(L108I)3x mice are transmissible to mice expressing the L108I variant but not to wild‐type mice

To validate that the spontaneous neurodegenerative condition in TgMo(L108I)3x mice constitutes an authentic prion disorder, we examined its capacity to fulfill a critical diagnostic criterion: the generation of transmissible pathogenic agents capable of inducing similar disease when introduced into new hosts. This transmissibility represents a fundamental property distinguishing prion disorders from other neurodegenerative conditions.

We conducted intracerebral inoculations using brain homogenates from symptomatic TgMo(L108I)3x mice into a panel of recipient models with varying PrP configurations. These models were categorized according to whether they expressed the WT L108 variant or the L108I variant of mouse PrP, and at what expression levels. Among recipients with WT L108 PrP, we observed a clear influence of expression level on susceptibility. Standard C57BL/6 mice showed complete resistance to transmission, with no animals developing clinical manifestations upon inoculation with brain homogenates from three different ill TgMo(L108I)3x mice despite extended monitoring periods (440–650 dpi), as detailed in Table 1. Secondary passage attempts using brain material from these mice similarly yielded negative results after more than 500 dpi, indicating a robust transmission barrier. Conversely, Tga20 mice, which express WT mouse PrP at six to eight times normal levels, developed characteristic neurological dysfunction with an average incubation period of 261 ± 16 days. Second passage in the same line showed modest acceleration to 230 ± 6 days. This efficient propagation in Tga20 mice is consistent with this model's well‐documented ability to propagate atypical strains, as GSS strain that fail to transmit in WT mice [23]. The high susceptibility conferred by PrP overexpression in Tga20 mice is well‐established and has proven capable of propagating strains unable to transmit in 1× expression models. Biochemical evaluation of brain tissue from these mice confirmed the presence of the distinctive 7–10 kDa fragment characteristic of atypical prionopathies (Figure S6).

**TABLE 1:** Inoculation of spontaneously ill TgMo(L108I)3x brain homogenate in different mouse models expressing wild‐type (WT) murine PrP (L108) or L108I murine PrP variant.

Animal model	Inoculum	Passage	Attack rate	Incubation period (dpi ± SEM)	PrP^res^ atypical pattern^a^ (Western blot)
WT mice (C57BL/6)	TgMo(L108I)3x‐01	First	0/6	>500	0/6
	TgMo(L108I)3x‐02	First	0/6	>440	0/6
	TgMo(L108I)3x‐03	First	0/6	>500	0/6
	WT‐TgMo(L108I)3x‐01	Second	0/5^b^	>500	0/5
	WT‐TgMo(L108I)3x‐02	Second	0/7	>500	0/7
	WT‐TgMo(L108I)3x‐03	Second	0/7	>500	0/7
Tga20	TgMo(L108I)3x‐02	First	7/7	261 ± 16	7/7
	Tga20‐TgMo(L108I)3x‐02	Second	6/6	230 ± 6	6/6
TgMo(L108I)3x	TgMo(L108I)3x‐01	First	7/7	105 ± 8	7/7
TgMo(L108I)1x	TgMo(L108I)3x‐01	First	6/6	480 ± 31	6/6

Abbreviations: dpi, days post‐inoculation; SEM, standard error of the mean.

^a^ PrP^res^ atypical pattern (Western blot): refers to the detection of a low molecular weight PK‐resistant fragment by Western blot, as detailed in Section 2.

^b^ Originally the group was composed of 7 mice, but two were culled (at 155 and 338 dpi) because of intercurrent diseases.

In stark contrast to the expression level‐dependent transmission observed in WT L108 models, recipients expressing the L108I variant demonstrated markedly enhanced susceptibility regardless of expression levels. TgMo(L108I)3x mice exhibited full susceptibility upon intracerebral inoculation with accelerated disease onset at 105 ± 8 dpi. TgMo(L108I)1x mice, expressing the variant at physiological levels, also developed disease but with extended incubation periods, demonstrating that while the polymorphism facilitates transmission, PrP concentration significantly modulates propagation kinetics. Both models displayed atypical biochemical profiles featuring the signature low molecular weight bands upon analysis (Figure S6). These transmission studies demonstrate that the spontaneously arising neurological condition in TgMo(L108I)3x mice produces authentic infectious agents that can efficiently propagate disease in appropriate host models, particularly those expressing identical PrP variants or those with elevated PrP levels. This transmissibility confirms the genuine prion nature of the spontaneous disorder in this transgenic model.

### 3.6 Efficient transmission of atypical and classical prion strains in the TgMo(L108I) models

Given the predisposition of the TgMo(L108I)3x model to spontaneously generate an atypical prion strain, we evaluated its susceptibility to propagate other atypical strains, even from different species. We selected several well‐characterized atypical strains, including A117V GSS from a Spanish patient, the atypical ovine Nor98 strain (characterized by an atypical protease resistance pattern and PrP^res^ deposits primarily in the cerebellar and cerebral cortices), the spontaneously generated strain in the TgShI112 model (which develops a spontaneous prion disease with features indistinguishable from atypical Nor98 scrapie), and prions derived from the TgVole(I109)4x model [7]. Intracerebral inoculations of the TgMo(L108I)3x model demonstrated extraordinary susceptibility, with remarkably short incubation periods: 57 ± 0.6 days for A117V GSS, 114 ± 5.1 days for Nor98, 83 ± 1.9 days for TgShI112‐derived prions, and 76 ± 1.4 days for TgVole(I109)4x model prions (Table 2). The efficiency and rapidity of propagation of the A117V GSS strain are particularly striking, as it also infected efficiently via the intraperitoneal route (57 ± 1.4 days), with times virtually identical to those obtained after intracerebral inoculation. This contrasts markedly with the traditionally reported low infectivity of GSS in conventional experimental systems. Biochemical analysis of brain tissue from all affected animals revealed the expected pattern, showing the characteristic 7–10 kDa band typical of atypical prion diseases (Figure S7).

**TABLE 2:** Inoculation of TgMo(L108I)3x mice with different atypical prion strains.

Animal model	Inoculum	Inoculation route^a^	Passage	Attack rate	Incubation period (dpi ± SEM)	PrP^res^ atypical pattern^b^ (WB)
TgMo(L108I)3x	A117V GSS	i.c.	First	7/7	57 ± 0.6	7/7
			Second	7/7	73 ± 1	7/7
		i.p.	First	6/6	57 ± 1.4	6/6
	Nor98	i.c.	First	8/8	114 ± 5.1	8/8
	TgShI112‐Spon.	i.c.	First	7/7	83 ± 1.9	7/7
	TgVole(I109)4x‐Spon.	i.c.	First	8/8	76 ± 1.4	8/8
TgMo(L108I)1x	A117V GSS	i.c.	First	7/7	>500^c^	7/7
		i.p.	First	7/7	>500^c^	7/7

Abbreviations: dpi, days post‐inoculation; SEM, standard error of the mean.

^a^ i.c.: intracerebral inoculation of 1% brain homogenate; i.p.: intraperitoneal inoculation of 10% brain homogenate.

^b^ PrP^res^ atypical pattern (WB): refers to the detection of a low molecular weight PK‐resistant fragment by Western blot, as detailed in Section 2.

^c^ Animals from these two groups were culled without any sign of neurological impairment because of experiment termination at 502 dpi. However, the biochemical analysis of their brain homogenate showed PrP^res^ with atypical features, indicating the presence of a subclinical prion infection. For this reason, despite the lack of clinical signs, attack rate was considered 100%.

To examine the effect that the amount of PrP L108I has on the propagation of atypical prions, we performed intracerebral and intraperitoneal inoculations of the TgMo(L108I)1x model with A117V GSS. Surprisingly, none of the seven animals per group developed the disease, although biochemical analysis revealed the presence of the typical 7–10 kDa band, demonstrating that the prion had propagated without manifesting clinical signs (Table 2 and Figure S6). This dissociation between prion propagation and clinical disease reveals a critical threshold effect: while the L108I substitution facilitates efficient prion conversion at physiological expression levels, clinical manifestation requires additional PrP expression to overcome cellular tolerance mechanisms.

Having established the model's exceptional capacity for atypical prions, we next evaluated its performance with well‐established classical prion strains. The TgMo(L108I)3x model showed efficient propagation with incubation periods of 85 ± 3.8 days for RML, 95 ± 1 days for 22L (previously reported [17]), 124 ± 3.9 days for 301C, and 212 ± 17.5 days for CWD‐TgVole. In the TgMo(L108I)1x model, the periods were 113 ± 1.5 days for RML and 167 ± 2.4 days for 22L. In C57BL/6 (WT) mice, the times were 169 ± 2.3 days for RML, 168 ± 1.4 days for 22L, and 184 ± 1.7 days for 301C (Table 3 and Figure S8). Consistent with Figure S8, PK–Western blot analysis of brains from all affected animals inoculated with RML, 22L, or 301C showed the canonical three‐band PrP^res^ pattern indistinguishable from the corresponding inocula across TgMo(L108I)3x, TgMo(L108I)1x, and WT recipients, indicating preservation of each strain's biochemical signature during propagation.

**TABLE 3:** Intracerebral inoculation of TgMo(L108I)3x mice with different classical prion strains.

Animal model	Inoculum	Attack rate	Incubation period (dpi ± SEM)	PrP^res^ classical pattern^a^ (WB)
TgMo(L108I)3x	RML^b^	5/5	85 ± 3.8	5/5
	22L^b^	8/8	95 ± 1	8/8
	301C	7/7	124 ± 4	7/7
	CWD‐TgVole	6/6	212 ± 17	6/6
TgMo(L108I)1x	RML	6/6	113 ± 1	6/6
	22L	5/5	167 ± 2	5/5
WT mice (C57BL/6)	RML	9/9	169 ± 2	9/9
	22L	9/9	168 ± 1	9/9
	301C	9/9	184 ± 2	9/9

Abbreviations: dpi, days post‐inoculation; SEM, standard error of the mean; WT, wild‐type.

^a^ PrP^res^ classical pattern (WB): refers to the detection of the classical three‐banded PK‐resistant fragment pattern by Western blot after PK digestion, as detailed in Section 2.

^b^ The results from these inoculations were already published in a previous manuscript from the group [17].

Importantly, secondary transmission experiments performed previously demonstrated that classical prion strains maintain their fundamental properties after passage through the TgMo(L108I)3x model. When RML and 22L strains that had been passaged through TgMo(L108I)3x mice were subsequently inoculated into WT mice, they displayed incubation periods and biochemical patterns indistinguishable from the original strains [17]. To confirm the conservation of their biological properties, a detailed histopathological analysis was carried out. As shown in Figure S9, lesion profiles of RML and 22L in WT (C57BL/6) mice and TgMo(L108I)3x‐adapted RML and 22L when back‐passaged to WT mice are almost identical in terms of shape, spongiform lesions and PrP^res^ deposits occurring in the same brain areas. Although these present with lower intensity in the back‐passaged strains, mainly in cortical areas and hippocampus, indicating an overall conservation of the lesion distribution pattern and thus, the main biological features of each strain. This preservation of strain characteristics indicates that neither the L108I substitution nor the overexpression fundamentally alters the core properties of classical prion strains, consistent with previous observations in Tga20 mice expressing WT PrP at high levels [24].

## 4 DISCUSSION

Our complementary transgenic mouse models expressing mouse L108I PrP presented here provide important insights into the role of this polymorphism in spontaneous prion formation and strain‐specific propagation. By employing both traditional random insertion transgenic methods and precise CRISPR‐Cas9 genome editing, we have successfully dissected the relative contributions of sequence alteration and PrP^C^ expression level to the development of spontaneous prion disease.

The TgMo(L108I)3x overexpression model clearly demonstrates that the L108I substitution, when expressed at approximately three times normal levels, is sufficient to induce spontaneous prion disease with characteristics mirroring atypical prionopathies such as Nor98 in sheep [9] and GSS in humans [5]. However, the knock‐in TgMo(L108I)1x model, expressing the same variant at physiological levels, remained free of spontaneous disease despite extended monitoring periods exceeding 600 days. This striking contrast provides compelling evidence that both factors—the specific amino acid substitution and elevated expression levels—are necessary for spontaneous prion formation in this system.

Our findings align with previous observations in related models, particularly the work by Watts et al. with bank vole PrP [4], where expression level significantly influenced disease onset timing. Similarly, our own work with transgenic models expressing bank vole PrP^C^ with the I109 polymorphism at different expression levels leads to the same conclusion [7]. The I109 position in bank vole PrP (corresponding to I108 in our mouse models) appears to create an inherent structural vulnerability that enhances misfolding propensity, yet requires a threshold concentration of protein to trigger spontaneous disease within the lifespan of mice. Importantly, Tga20 mice expressing WT mouse PrP at six to eight times normal levels [25] do not develop spontaneous disease during their natural lifespan, confirming that the L108I substitution—not merely PrP overexpression—is the primary driver of spontaneous pathogenesis. Recent comprehensive studies have further demonstrated that while PrP overexpression can lead to age‐dependent proteinopathy in very aged animals, this pathology is critically non‐transmissible, contrasting with the authentic infectious prions generated in our TgMo(L108I)3x model [26].

Unlike other spontaneous prion models such as TgVole(I109)4x [7], we observed no sex‐dependent differences in disease onset or progression in the TgMo(L108I)3x model. This finding suggests that while the isoleucine substitution is the primary driver of spontaneous prion formation, the specific host context (mouse vs. bank vole PrP) may influence the impact of biological variables such as sex on disease kinetics.

The biochemical and neuropathological features of the spontaneous disease in TgMo(L108I)3x mice strongly resemble those of atypical prion disorders. The characteristic low molecular weight band (7–10 kDa) following PK digestion and the distribution of PrP^res^ deposits—predominantly in Cc, neocortex and thalamic nuclei, and with a characteristic hippocampal involvement including CA1 atrophy, pyramidal neuron loss and gliosis (Figure 2D)—mirrors patterns observed in naturally occurring atypical prionopathies [27, 28, 29]. These features contrast with the brainstem‐predominant pathology typically seen in classical prion diseases, further supporting the classification of this spontaneous disease as an atypical prion disorder.

The convergence of similar disease phenotypes across different species when isoleucine is introduced at equivalent positions (108 in mice, 109 in bank voles [7] 112 in sheep [6]) suggests a conserved mechanism underlying spontaneous prion formation. Understanding of the key molecular features that enable swift and consistent spontaneous prion generation has revealed common structural determinants that facilitate the transition from cellular to pathological prion protein conformations [30]. Sigurdson et al. demonstrated that even subtle alterations to the structure of the β2‐α2 loop region can drive spontaneous prion formation in mice [31]. Their findings, like ours, emphasize how minimal changes to PrP structure can fundamentally alter its propensity for pathological folding and aggregation.

Our transmission studies provide compelling evidence that TgMo(L108I)3x mice generate authentic, transmissible prions. A notable finding is the existence of a robust transmission barrier to WT mice expressing physiological levels of PrP. Standard C57BL/6 mice showed complete resistance to transmission, with no animals developing clinical manifestations despite extended monitoring periods. However, this transmission barrier could be partially overcome in Tga20 mice, which express WT mouse PrP at six to eight times the normal levels. This represents the first documented instance of a mouse model expressing WT mouse PrP (L108) exhibiting an atypical PrP^Sc^ pattern following inoculation.

For models expressing the I108 variant, transmission efficiency was markedly enhanced. This clear distinction in transmission efficiency based on the presence of isoleucine at position 108 highlights the critical role of specific amino acid identity in facilitating prion propagation, independent of expression level. The homologous nature of the PrP sequence between donor and recipient appears to be the primary determinant of efficient transmission, while PrP concentration acts as a secondary modulator of propagation kinetics. The critical role of position 108 in prion susceptibility has been previously demonstrated through comparative studies of mouse models with *Prnp*(a) (L108) and *Prnp*(b) (F108) genotypes [15, 16]. In these studies, the L108 variant consistently showed greater susceptibility to various scrapie isolates, further validating the importance of this residue in prion transmission.

A critical finding from our transmission studies is the existence of a minimum threshold of PrP expression required for clinical manifestation of prion disease, despite evidence of subclinical propagation. This was clearly demonstrated with A117V GSS inoculations into TgMo(L108I)1x mice, where none of the animals developed clinical disease, yet biochemical analysis revealed the characteristic 7–10 kDa band indicative of prion propagation. This dissociation between propagation and clinical manifestation provides a valuable experimental system for studying the relationship between prion amplification and neurotoxicity. It also offers opportunities to evaluate therapeutic approaches targeting prion‐induced pathology. Alternatively, these animals could be propagating a PrP amyloid unable to cause prion disease, as observed previously for murine models expressing GSS‐related mutations [32]. However, the behavior of the overexpressing I108 model upon GSS inoculation and the susceptibility of TgMo(L108I)1x mice to spontaneous TgMo(L108I)3x prions suggest that the knock‐in mice develop subclinical disease with signs that do not manifest during their lifespan because of slower propagation kinetics associated with lower PrP expression levels.

Beyond these threshold effects, the differential impact of the L108I substitution on the propagation of distinct classical prion strains further evidences the complex interplay between prion strain properties and host PrP sequence. When comparing animals with equivalent levels of PrP expression, the isoleucine substitution produces a markedly more significant reduction in the incubation period for RML (~33% shorter) than for 22L (~0.5% shorter) compared to WT mice. This strain‐specific effect is maintained when PrP expression is increased to 3x (Table 3), with proportional reductions in incubation times. This strain‐selective effect echoes the classic *Prnp*(a)/*Prnp(*b) paradigm in congenic mice, where ME7, RML, and 139A transmit rapidly in *Prnp*(a) (L108/T189; e.g., C57BL, RIII) but slowly in *Prnp*(b) (F108/V189; VM), whereas 22A and 87V show the opposite pattern [15, 33, 34].

Particularly striking is the observation that incubation times between TgMo(L108I)3x and Tga20 mice are comparable for classical strains like RML and 22L. Comparing with published data for Tga20 mice [24], which express WT mouse PrP at six to eight times normal levels (with incubation periods of 59 ± 8 days for RML and 83 ± 3 days for 22L according to Karapetyan et al.), we observe similar incubation times in our TgMo(L108I)3x model. This equivalence suggests that the effect of the isoleucine substitution at position 108 is substantial, allowing similar acceleration of prion propagation with lower expression levels. In essence, rapid propagation times can be achieved either with 6–8× expression of mouse L108 PrP or with 3× expression of mouse I108 PrP.

Importantly, secondary transmission experiments with RML and 22L passaged through TgMo(L108I)3x mice back to WT mice demonstrated incubation periods, biochemical patterns and lesion profiles almost identical to the original strains [17] (Figures S5 and S6), confirming that neither the isoleucine substitution nor the overexpression fundamentally alters the core properties of these classical strains.

The transmission characteristics of A117V GSS in the TgMo(L108I)3x model reveal a particularly intriguing phenomenon: Nonadaptive Prion Amplification (NAPA), as described by Bian et al. [35]. The exceptionally rapid transmission in the first passage (57 ± 0.6 days) followed by a slightly prolonged incubation period in the second passage (73 ± 1 days) runs counter to the typical pattern of strain adaptation, where incubation periods typically shorten upon serial passage. In NAPA, prions replicate in a new host without fully adapting their conformational properties, maintaining characteristics of the original strain. This interpretation is further supported by the complete transmission barrier observed when attempting to passage these prions to WT mice—none of the six animals developed disease even after more than 600 dpi, indicating that the A117V GSS prions maintained their original structural characteristics. These results highlight the low barrier or even the possible NAPA effect in relation to the efficiency and rapidity with which the human atypical strain infects and propagates in the TgMo(L108I)3x model.

Beyond its utility for propagating naturally occurring prion strains, the TgMo(L108I)3x model has also proven valuable for investigating recombinant prions, as demonstrated in recent work by Pérez‐Castro et al. [17] Their study showed that this model efficiently propagates recombinant prions generated in vitro, including those formed both in the presence and absence of polyanionic cofactor [17].

In conclusion, the TgMo(L108I) models presented here provide complementary, versatile tools for prion research. The higher‐expression TgMo(L108I)3x line shows exceptional sensitivity for propagating diverse prion strains, including atypical conformers that have traditionally been difficult to transmit. In contrast, the TgMo(L108I)1x line enables investigation of sequence effects independent of overexpression. Together, these models advance our understanding of the molecular determinants of prion‐strain formation, selection, and transmissibility, with implications for fundamental biology and therapeutic development.

## AUTHOR CONTRIBUTIONS

**HE**, **EV**, **NF‐B**, and **JC**: Conceptualization, methodology, formal analysis, writing—original draft, writing—review and editing, supervision, funding acquisition. **JMC**, **CMD‐D**, **CS‐T‐Q**, **JG‐A**, **EF‐M**, **MS‐J‐A**, **MAP‐C**, **NG‐A**, **PP**, **SG**, **NG‐M**, **NLL**, and **AM‐A**: Methodology, investigation, formal analysis. **GPdN** and **MG**: Methodology, investigation, formal analysis, writing—review and editing. **MAS‐M** and **JRR**: Methodology, investigation, resources, writing—review and editing, funding acquisition.

## FUNDING INFORMATION

The present work was partially funded by three different grants awarded by “Agencia Estatal de Investigación, Ministerio de Ciencia e Innovación” (Spanish Government), grant numbers PID2024‐160022OB‐I00, PID2021‐122201OB‐C21 granted to Joaquín Castilla, PID2020‐117465GB‐I00 granted to Jesús R. Requena, and PID2021‐1222010B‐C22 granted to Enric Vidal, funded by MCIN/AEI/10.13039/501100011033 and co‐financed by the European Regional Development Fund (ERDF). EFA031/01 NEURO‐COOP, which is co‐funded at 65% by the European Union through Programa Interreg VI‐A España‐Francia‐Andorra (POCTEFA 2021–2027) granted to Joaquín Castilla, Hasier Eraña and Enric Vidal. Additionally, CIC bioGUNE currently holds a Severo Ochoa Excellence accreditation, CEX2021‐001136‐S granted to Joaquín Castilla, also funded by Ministerio de Ciencia e Innovación/AEI/10.13039/501100011033. Transgenic Facility of IBSAL‐University of Salamanca, directed by Manuel A. Sánchez‑Martín, is supported by Instituto de Salud Carlos III (ISCIII), co‐funded by the European Union grant PT23/00123. Eva Fernández‐Muñoz received funding from Fundación Tatiana Pérez de Guzmán el Bueno, grant BN661‐FTPGB‐2023. This work also received funding from the Creutzfeldt‐Jakob Disease Foundation—2022. The funders had no role in study design, data collection and analysis, decision to publish, or preparation of the manuscript.

## CONFLICT OF INTEREST STATEMENT

Authors Hasier Eraña and Jorge M. Charco are employed by the commercial company ATLAS Molecular Pharma SL. This does not alter our adherence to the Journal's policies on sharing data and materials and did not influence in any way the work reported in this manuscript, given that the company had no role in study design, funding, and data analysis. The rest of the authors declare no competing interests.

## Supporting information


**Data S1.** Supporting Information.

## Data Availability

The datasets generated and analyzed during the current study are available from the corresponding author upon request. This includes raw histopathological images, Western blot data, survival analysis datasets, and biochemical analysis results. Access to transgenic mouse lines TgMo(L108I)3x and TgMo(L108I)1x may be provided through material transfer agreements subject to institutional approvals and ethical considerations.
