# Supplementary material for: The L108I polymorphism in mouse prion protein drives spontaneous disease and enhances transmission of atypical and classical prion strains
Source: Brain Pathol. 2026 Feb 9;36(4):e70083. doi: 10.1111/bpa.70083 (PMC13238759; doi:10.1111/bpa.70083)
Supplement: Supplementary file 1 — Data S1. Supporting Information. [file BPA-36-e70083-s001.docx]

**Supplementary Material**

**The L108I polymorphism in mouse prion protein drives spontaneous disease and enhances transmission of atypical and classical prion strains**

Running Title

**L108I polymorphism drives prion disease**

Hasier Eraña^1,2,3^, Enric Vidal^4,5,*^, Natalia Fernández-Borges^1,*^, Jorge M. Charco^1,2,3^, Carlos M. Díaz‑Domínguez^1^, Cristina Sampedro-Torres-Quevedo^1,2^, Josu Galarza-Ahumada^1^, Eva Fernández-Muñoz^1^, Maitena San-Juan-Ansoleaga^1^, Miguel Ángel Pérez-Castro^1^, Nuno Gonçalves-Anjo^1^, Patricia Piñeiro^1^, Samanta Giler^4,5^, Nora González-Martín^6^, Nuria L. Lorenzo^7^, Africa Manero-Azua^8^, Guiomar Perez de Nanclares^8^, Mariví Geijo^9^, Manuel A. Sánchez‑Martín^6,10^, Jesús R. Requena^7^, and Joaquín Castilla^1,2,11,#^

^1^ Center for Cooperative Research in Biosciences (CIC BioGUNE), Basque Research and Technology Alliance (BRTA), Derio, Spain.

^2^ Centro de Investigación Biomédica en Red de Enfermedades infecciosas (CIBERINFEC), Carlos III National Health Institute, Madrid, Spain.

^3^ ATLAS Molecular Pharma S. L., Derio, Spain.

^4^ IRTA. Programa de Sanitat Animal. Centre de Recerca en Sanitat Animal (CReSA). Campus de la Universitat Autònoma de Barcelona (UAB), Bellaterra, Catalonia. Spain.

^5^ Unitat mixta d’Investigació IRTA-UAB en Sanitat Animal. Centre de Recerca en Sanitat Animal (CReSA). Campus de la Universitat Autònoma de Barcelona (UAB), Bellaterra, Catalonia. Spain.

^6^ Institute for Biomedical Research of Salamanca (IBSAL), Salamanca, Spain.

^7^ CIMUS Biomedical Research Institute, University of Santiago de Compostela-IDIS, Santiago, Spain.

^8^ Molecular (Epi)Genetics Laboratory, Bioaraba Health Research Institute, Araba University Hospital, Vitoria-Gasteiz, Spain.

^9^ Animal Health Department, NEIKER-Basque Institute for Agricultural Research and Development. Basque Research and Technology Alliance (BRTA), Derio, Spain.

^10^ Transgenic Facility. Department of Medicine, University of Salamanca, 37007 Salamanca, Spain.

^11^ IKERBASQUE, Basque Foundation for Science, Bilbao, Spain.

Hasier Eraña herana.atlas@cicbiogune.es

Enric Vidal* enric.vidal@irta.cat

Natalia Fernández-Borges* natalia.fernandez@inia.csic.es

Jorge M. Charco jmoreno@cicbiogune.es

Carlos M. Díaz-Domínguez cdiaz@cicbiogune.es

Cristina Sampedro-Torres-Quevedo csampedro@cicbiogune.es

Josu Galarza-Ahumada jgalarza@cicbiogune.es

Eva Fernández-Muñoz efernandez@cicbiogune.es

Maitena San-Juan-Ansoleaga msanjuan@cicbiogune.es

Miguel Ángel Pérez-Castro mperez@lunenfeld.ca

Nuno Gonçalves-Anjo nanjo@cicbiogune.es

Patricia Piñeiro ppineiro@cicbiogune.es

Samanta Giler samanta.giler@irta.cat

Nora González-Martín noragonz@usal.es

Nuria L. Lorenzo nuria_2l_92@hotmail.com

Africa Manero-Azua africa.maneroruizdeazua@bio-araba.eus

Guiomar Perez de Nanclares guiomar.perezdenanclaresleal@osakidetza.eus

Mariví Geijo mgeijo@neiker.eus

Manuel A. Sánchez-Martín adolsan@usal.es

Jesús R. Requena jesus.requena@usc.es

Joaquín Castilla jcastilla@cicbiogune.es

* These authors contributed equally to this work

# Corresponding author: Joaquín Castilla


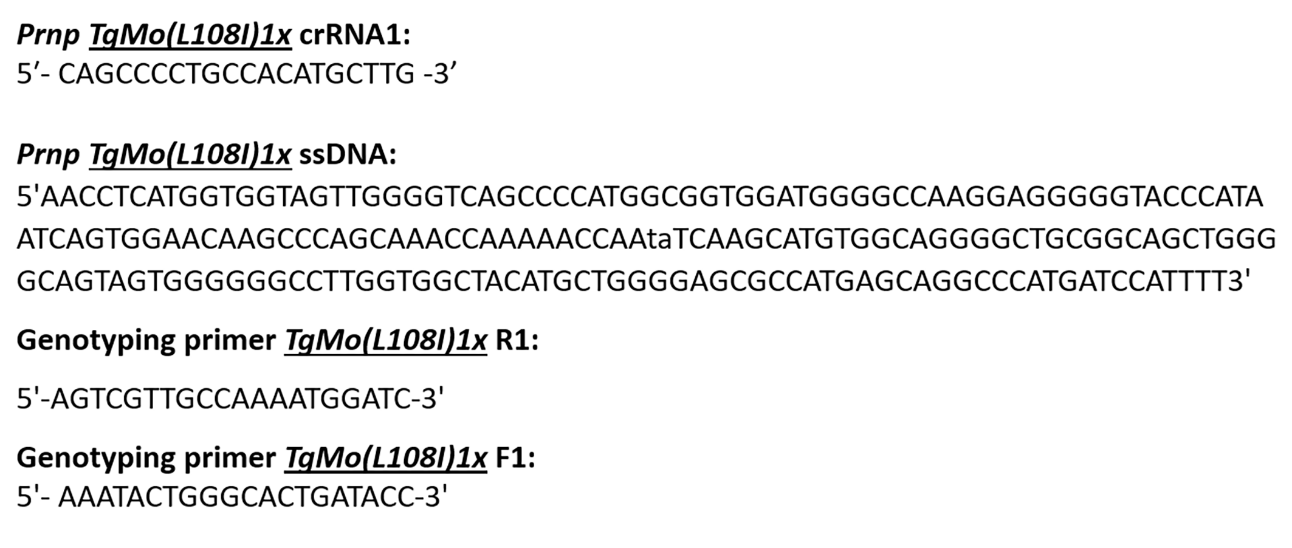
**Supplementary Figure 1. Molecular design for CRISPR-Cas9-mediated generation of TgMo(L108I)1x mice.** CRISPR-Cas9 components used for knock-in mouse generation: crRNA1 targeting *Prnp* exon 1, 200-nucleotide ssDNA template containing the c.322 C>A substitution (L108I), and genotyping primers (R1/F1) flanking the target region for founder validation.


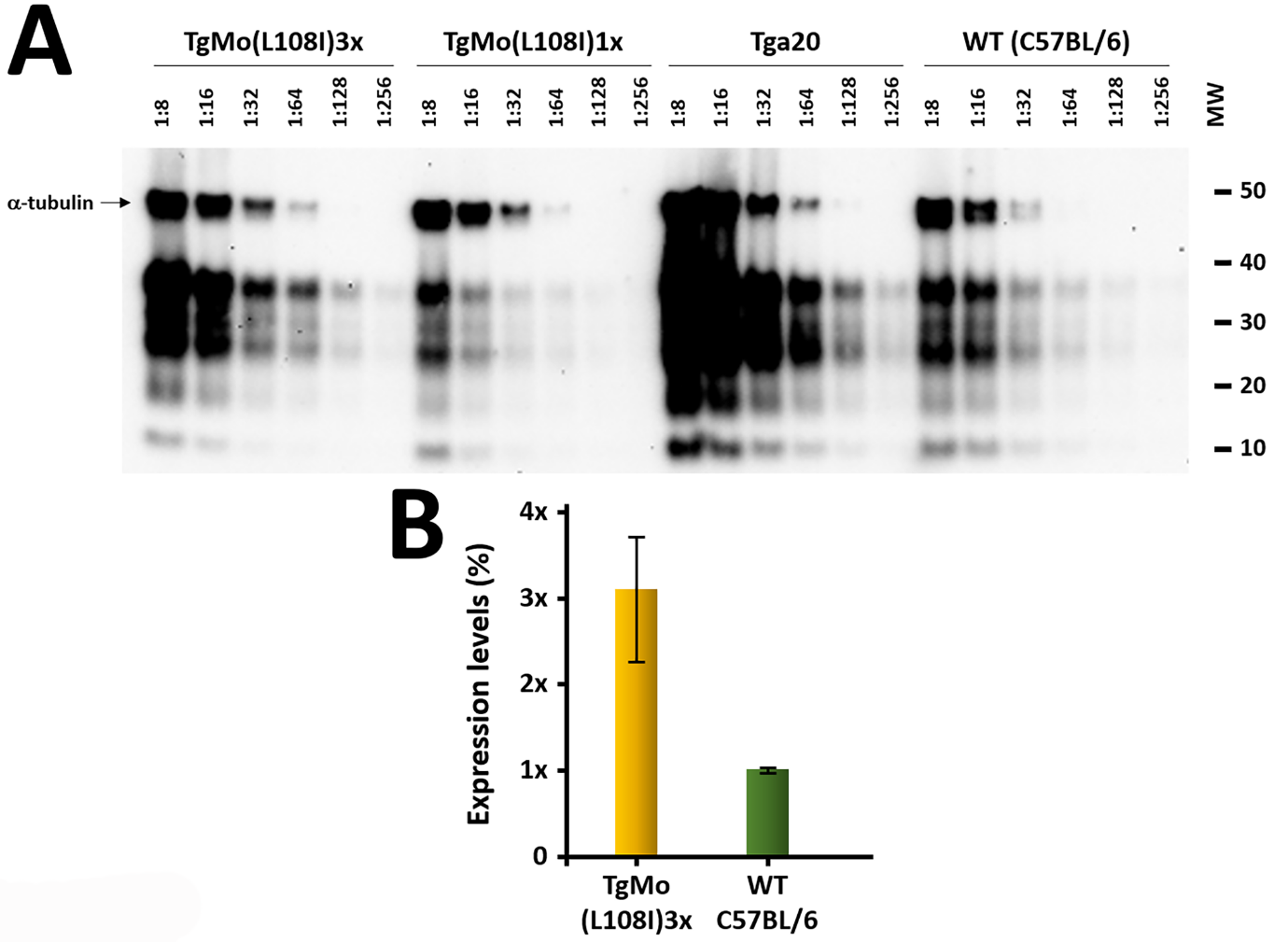


**Supplementary Figure 2. PrP^C^ expression levels in TgMo(L108I)3x and TgMo(L108I)1x mice compared to wild-type and Tga20 controls by Western blot.** **A)** PrP^C^ expression levels were assessed in the two new models: TgMo(L108I)3x mice (overexpressing L108I PrP via classical transgenesis) and TgMo(L108I)1x mice (a CRISPR-Cas9 knock-in expressing L108I PrP at physiological levels). Serial dilutions of 10% (w/v) brain homogenates were analyzed by Western blot using anti-PrP^C^ mAb Sha-31 (1:4,000). Brain homogenates from wild-type mice (C57BL/6 strain) and Tga20 mice, a previously established mouse model overexpressing approximately 8-fold wild-type mouse PrPC in the brain (Karapetyan et al. 2009), were used as controls at the same dilutions (1:8, 1:16, 1:32, 1:64, 1:128 and 1:256). The membrane was also probed with anti-α-tubulin mAb (1:8,000) as a loading control. The Western blot shows that the glycosylation pattern of PrP^C^ remains unaltered in the transgenic models compared to wild-type mice. **B)** Densitometric quantification of PrP^C^ expression levels from three independent brain samples per group confirms that TgMo(L108I)3x mice overexpress PrP^C^ at 3.14 ± 1.42-fold (mean ± SEM) compared to wild-type mouse brain (p < 0.001, Student's t-test), while the knock-in model TgMo(L108I)1x shows expression levels identical to wild-type mice.


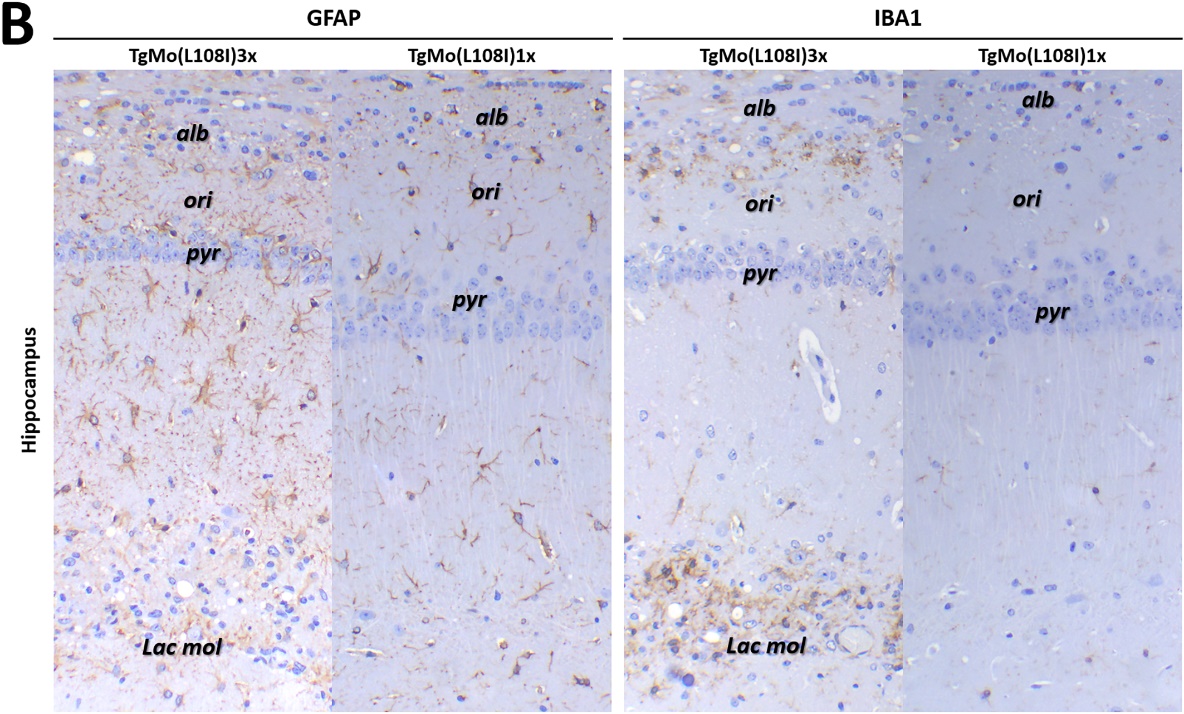

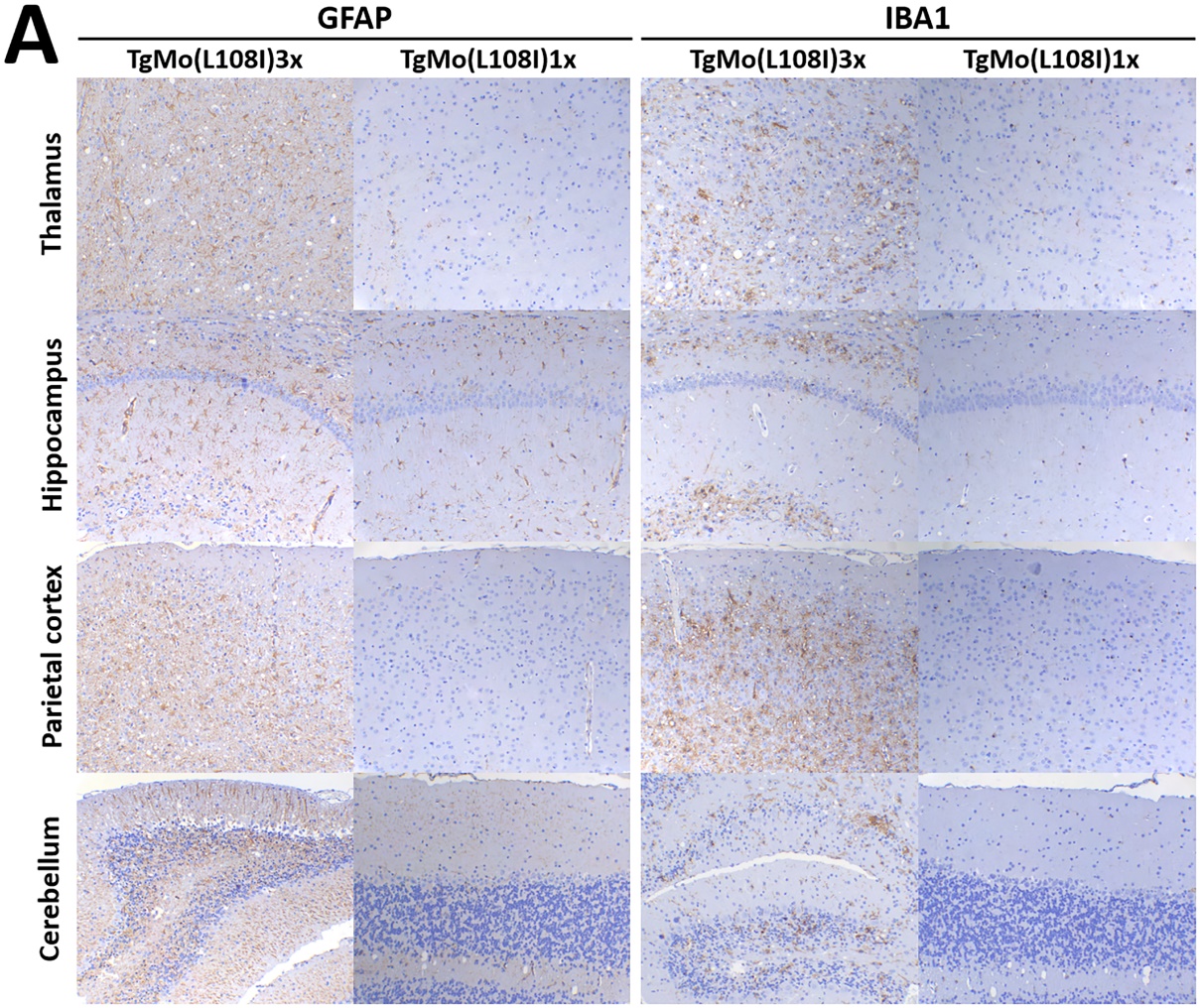


**Supplementary Figure 3: Comparative neuropathological analysis of glial activation in TgMo(L108I) transgenic mice.** Immunohistochemistry for glial fibrillary acidic protein (GFAP) to label astrocytes (left panels) and ionized calcium binding adapter molecule 1 (IBA1) to label microglia (right panels) in TgMo(L108I)3x mice that developed spontaneous pathology (left columns) compared to healthy TgMo(L108I)1x mice at 395 days of age (right columns). **A.** Distribution of gliosis across thalamus, hippocampus, parietal cortex, and cerebellum. **B.** Detail of glial activation in the hippocampus, showing stratum album (*alb*), stratum oriens (*ori*), stratum pyramidale (*pyr*), and stratum lacunosum-moleculare (*Lac mol*) layers. Animals with spontaneous phenotype exhibit intense astrogliosis and microgliosis characterized by cellular hyperplasia and hypertrophy, particularly prominent in neocortex (especially parietal cortex), hippocampus, thalamus, and cerebellar cortex. Hippocampal microgliosis was observed restricted to areas with PrP^res^ deposits. PrP^res^ plaques showed intense microglial infiltration but not astrocytic infiltration.


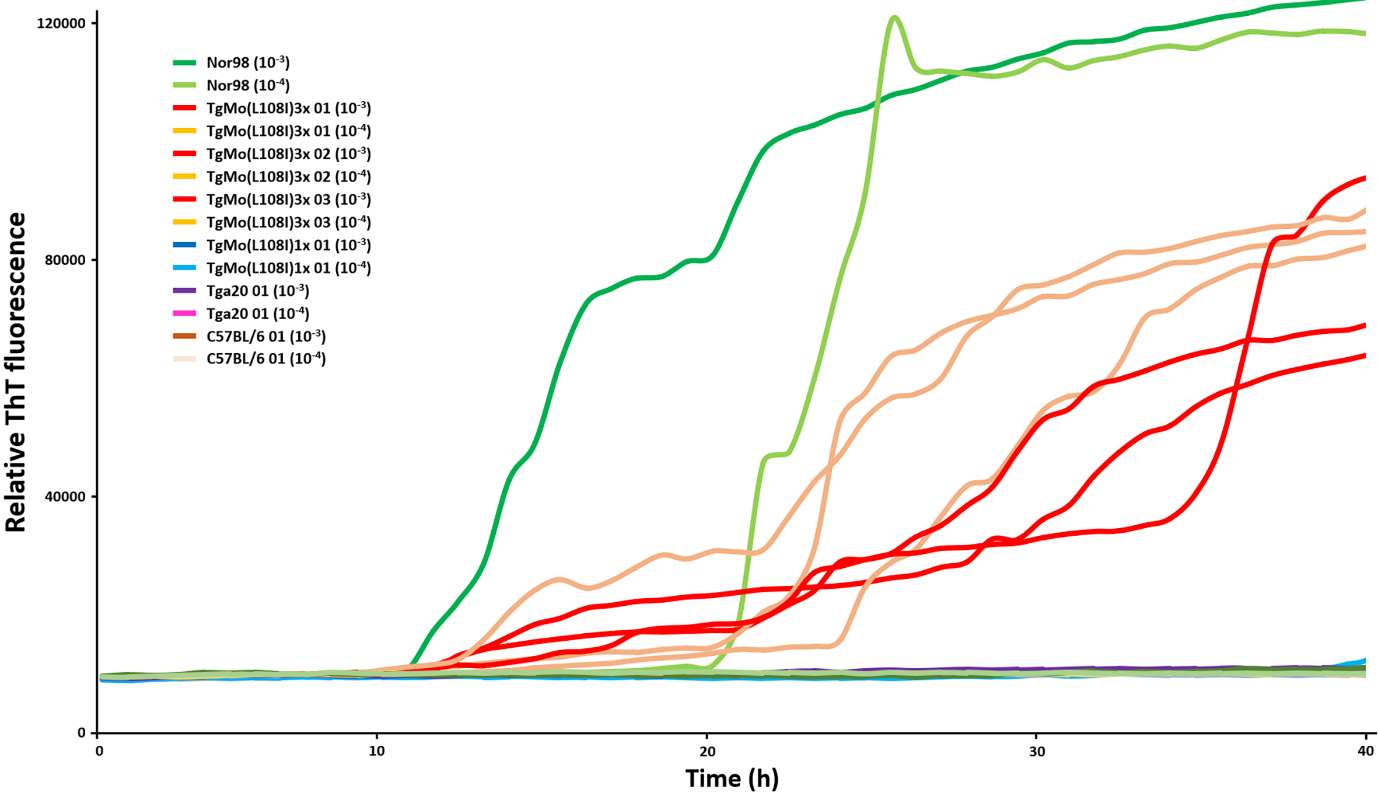


**Supplementary Figure 4. RT-QuIC analysis of brain extracts from different mouse models demonstrates seeding activity exclusively in TgMo(L108I)3x mice.** Brain homogenates from age-matched animals (~350 days old) were subjected to RT-QuIC analysis using recombinant bank vole I109 PrP as substrate. Brain extracts from three individual TgMo(L108I)3x mice with clinical signs (TgMo(L108I)3x 01, 02, and 03) showed robust seeding activity at both 10^-3^ and 10^-4^ dilutions (red and orange lines, respectively). Nor98 atypical scrapie served as positive control, demonstrating earlier onset kinetics at both dilutions (dark and light green lines for 10^-3^ and 10^-4^ dilutions, respectively). No seeding activity was detected in brain extracts from TgMo(L108I)1x (01), Tga20 (01), or wild-type C57BL/6 mice (01) at either 10^-3^ and 10^-4^ dilutions tested (flat lines at baseline: navy blue, purple, and brown/orange lines, respectively). The assay was performed in quadruplicate wells per sample, and representative curves are shown. This functional seeding data confirms that prion-conversion propensity is restricted to the TgMo(L108I)3x overexpression model and validates the presence of seeding-competent prions in the spontaneous disease.


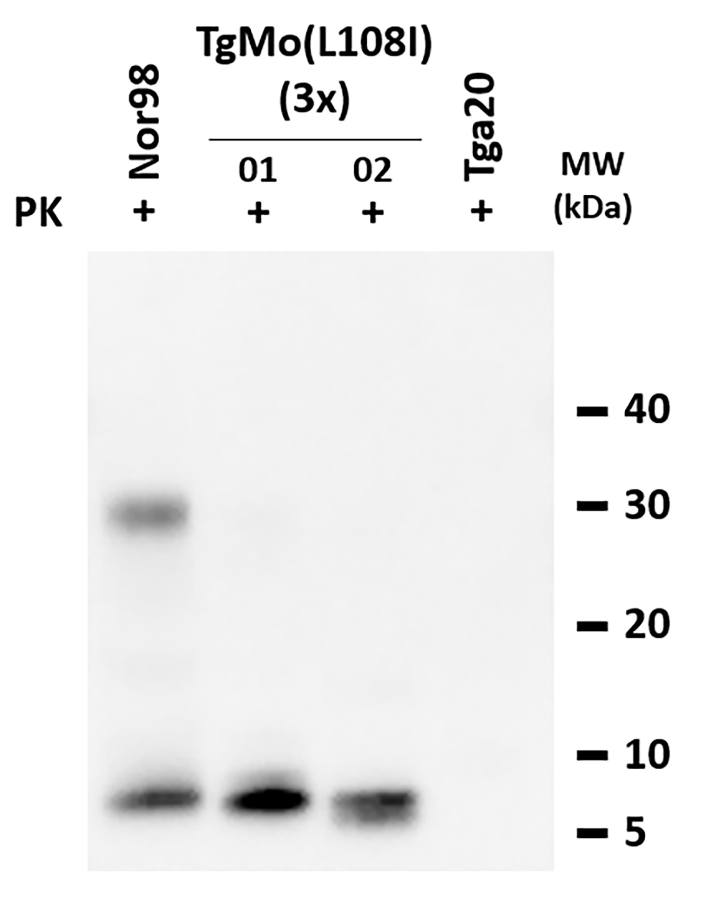


**Supplementary Figure 5. Biochemical comparison of aged mice from different overexpression models demonstrates atypical PrP^res^ exclusively in TgMo(L108I)3x mice.** Western blot analysis of proteinase K-resistant PrP from 10% (w/v) brain homogenates comparing spontaneously ill TgMo(L108I)3x mice (two representative animals culled at 334 and 357 days of age with clear signs of disease) and an aged Tga20 mouse (culled at 400 days of age showing no clinical signs). All samples were processed using the modified Wenborn protocol for atypical prion detection and digested with 10 μg/ml proteinase K (PK). The characteristic low molecular weight fragment (~7-10 kDa) is clearly visible in both TgMo(L108I)3x animals but completely absent in the aged Tga20 mouse, despite comparable or even higher PrP overexpression levels in the latter model. For comparison, atypical scrapie isolate (Nor98) processed identically is shown as positive control. Detection was performed using 9A2 monoclonal antibody (1:4,000). This biochemical analysis complements the RT-QuIC functional data (Supplementary Figure 4), demonstrating that seeding-competent atypical prions are generated exclusively in the L108I overexpression model and not in wild-type PrP overexpression alone. MW: molecular weight marker.

**
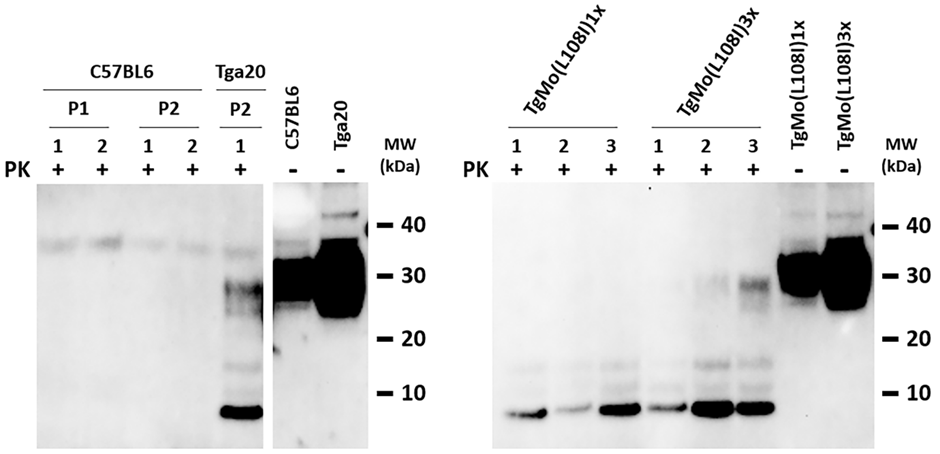
**

**Supplementary Figure 6. Detection of misfolded proteinase K resistant PrP in the distinct mouse models inoculated intracerebrally with spontaneously ill TgMo(L108I)3x brain homogenate.** To evaluate transmissibility of the spontaneous prion disease developed in TgMo(L108I)3x, different mouse models expressing either the wild type murine PrP (L108) (wild type mice, C57BL/6 and Tga20, overexpressing mouse PrP 6 to 8-fold) or the L108I variant (TgMo(L108I)3x and TgMo(L108I)1x) were inoculated intracerebrally and the presence of proteinase K-resistant misfolded PrP (PrP^res^) with atypical biochemical features was assessed upon culling due to signs of disease or experiment termination due to advanced age (>500 days post inoculation). The protocol developed by Wenborn and colleagues ^1^ was used to partially purify and detect the atypical PrP^res^, prior to electrophoresis and Western blotting (9A2 mAb, 1:4,000). Two representative examples of C57BL/6 mice (inoculated with isolate TgMo(L108I)3x-01, see table 1) are shown, that in agreement with the lack of neurological impairment signs are PrPres negative after more than 500 days post-inoculation (dpi) at passage 1 (P1) and even after secondary transmission (P2). In contrast, the Tga20 mice inoculated with the same brain homogenate presented the expected atypical PrP^res^ pattern as shown by a representative example from the second passage (P2). This PrP^res^ was also detected in the two models expressing L108I mouse PrP variant, as shown in the blot from the left, indicating that either the presence of isoleucine or the overexpression of the wild-type variant favour the transmission of this spontaneous prion disease. In each gel, undigested controls of the brain homogenate of each mouse line used are included to illustrate the differences in expression levels. PK: Proteinase K; MW: Molecular weight marker.


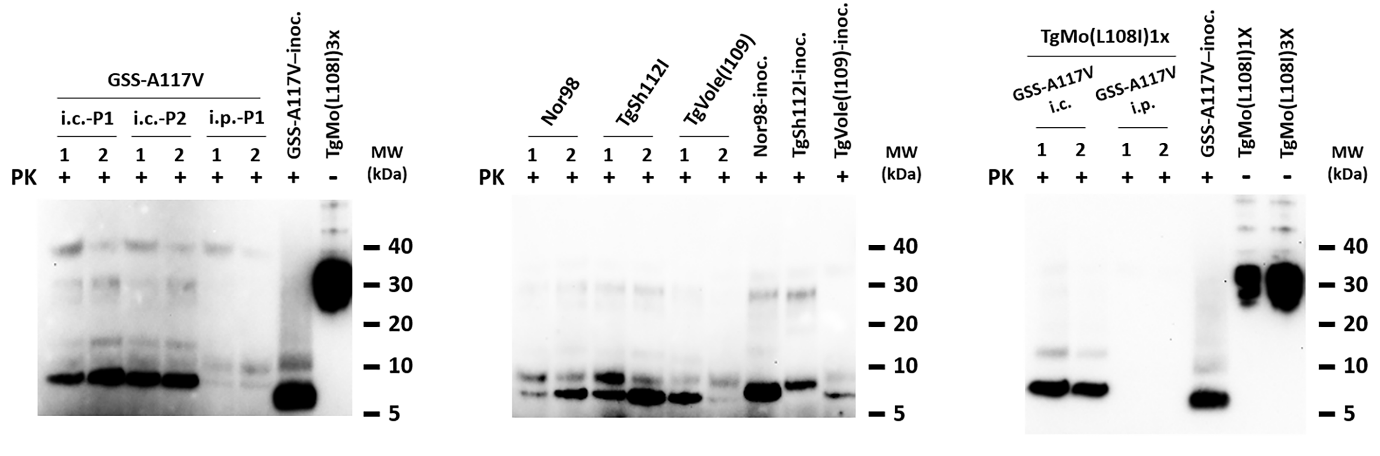
**Supplementary Figure 7. Detection of misfolded proteinase K resistant PrP in TgMo(L108I)3x and TgMo(L108I)1x mice inoculated with different atypical prion strains.** To evaluate the susceptibility of mouse L108I PrP expressing models to different atypical prion strains, TgMo(L108I)3x mice were inoculated with an A117V GSS isolate [two serial passages (P1 and P2) by intracerebral inoculation (i.c.) and one passage by intraperitoneal inoculation (i.p.)], a Nor98 atypical scrape isolate, TgSh112I spontaneous ovine atypical prion isolate ^2^ and spontaneous atypical prions formed in TgVole(I109)4x model; and TgMo(L108I)1x were also inoculated with the same A117V GSS isolate both intracerebrally and intraperitoneally, to evaluate the effect of the PrP^C^ expression levels on transmissibility. Brain homogenates of 2 representative animals from each group are shown. The presence of proteinase K-resistant misfolded PrP (PrP^res^) with atypical biochemical features was assessed upon culling due to signs of disease or experiment termination due to advanced age. The protocol developed by Wenborn and colleagues ^1^ was used to partially purify and detect the atypical PrP^res^, prior to electrophoresis and Western blotting (9A2 mAb, 1:4,000). All TgMo(L108I)3x mice, in agreement with their signs of neurological impairment present the expected atypical PrPres, with notably lower intensity in those infected intraperitoneally. In contrast, in TgMo(L108I)1x, that showed no sign of disease at the time of culling, present PrPres only in those inoculated intracerebrally. In the left and right gels, undigested controls of the brain homogenates of each mouse line used are included to illustrate the differences in expression levels and in all three gels, the original inoculums used for the bioassay are also shown (indicated by –inoc.). PK: Proteinase K; i.c.: intracerebral inoculation; i.p.: intraperitoneal inoculation; MW: Molecular weight marker.


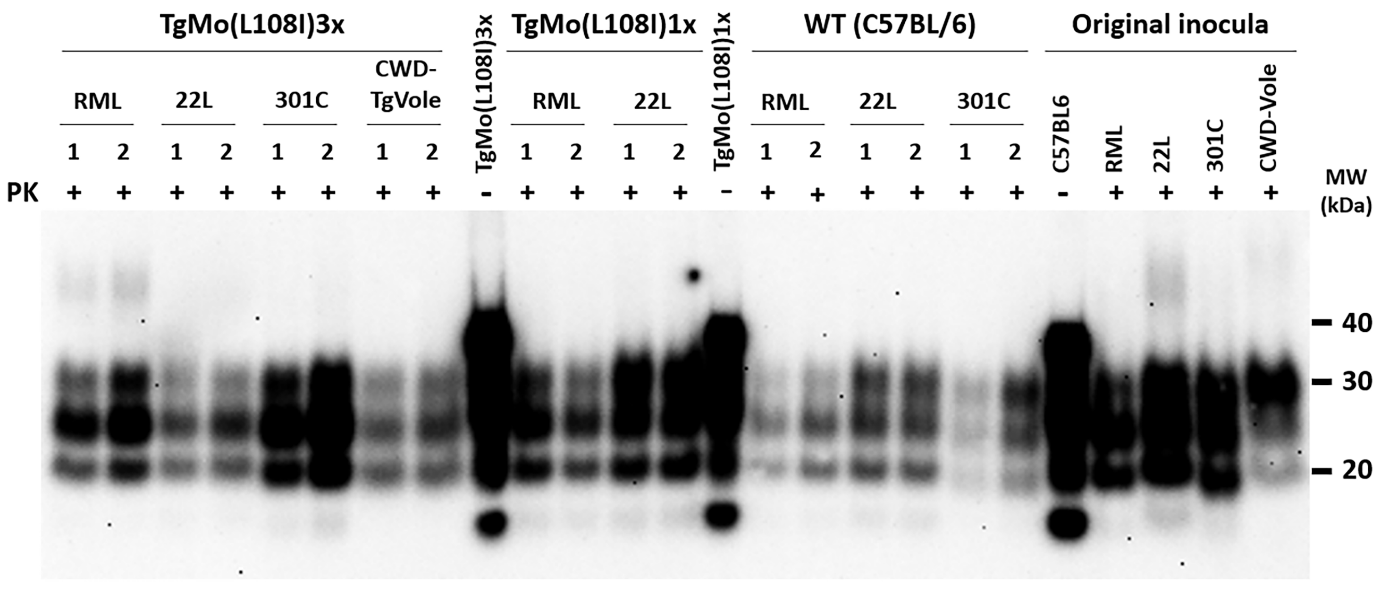
**Supplementary Figure 8. Detection of misfolded proteinase K resistant PrP in TgMo(L108I)3x, TgMo(L108I)1x and wild type (C57BL/6) mice inoculated with different classical prion strains.** To evaluate the susceptibility of mouse L108I PrP expressing models to different classical prion strains and compare their susceptibility and incubation periods with wild type mice, TgMo(L108I)3x mice were inoculated intracerebrally with the well-known murine prion strains RML, 22L and 301C and the TgVole-adapted CWD prion strain ^3^. TgMo(L108I)1x mice were inoculated intracerebrally with the same RML and 22L prions and as controls, wild type mice of the C57BL/6 strain were also inoculated intracerebrally with RML, 22L and 301C. Brain homogenates from two representative animals of each group are shown in the gel after proteinase K digestion, electrophoresis and Western blotting (Sha31 mAb, 1:4,000). Classical PrP^res^ exhibiting the characteristic three-banded pattern was detected in all analyzed animals, with undistinguishable features from the original inocula, included in the right part of the gel. Undigested controls of the brain homogenates of each mouse line used are included as a rapid size reference. PK: Proteinase K; MW: Molecular weight marker.


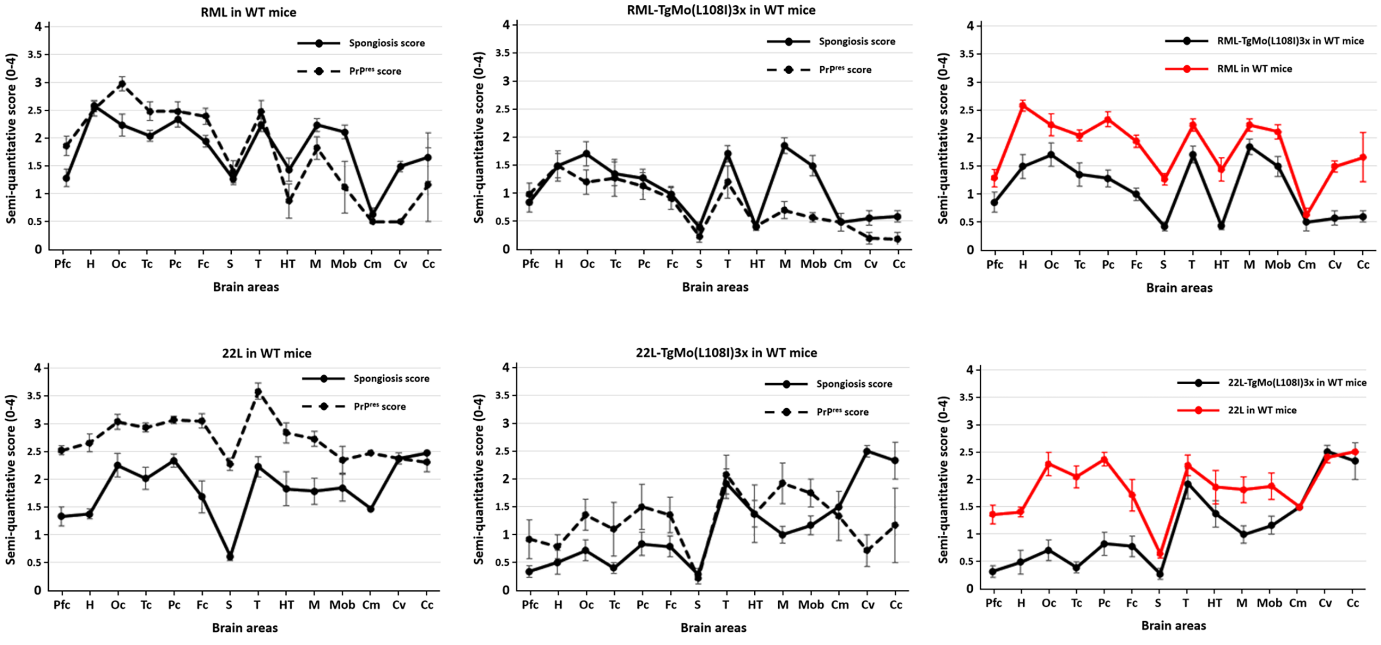
**Supplementary Figure 9. Semiquantitative (0 to 4) analysis of spongiform lesion and PrP^res^ deposition patterns in wild-type (C57BL/6) mice inoculated with RML and 22L compared to TgMo(L108I)3x-adapted RML and 22L back-passaged to wild-type mice, determined by histopathological and immunohistochemical analysis.** The left panels represent the spongiform lesion (solid black lines) and PrP^res^ deposition (dashed lines) profiles in wild-type (C57BL/6) mice inoculated intracerebrally with original RML and 22L strains. The center panels show the lesion profiles found upon intracerebral inoculation of TgMo(L108I)3x-adapted strains back-passaged to wild-type mice. The right panels show overlaid spongiform lesion profiles comparing wild-type mice inoculated with TgMo(L108I)3x-adapted strains (solid black lines) versus the original murine strains (solid red lines). The profiles demonstrate that TgMo(L108I)3x-adapted strains maintain characteristic lesion patterns similar to their original counterparts, although back-passaged strains show reduced lesion intensity, particularly in PrP^res^ deposits, possibly due to differences in titers. Brain regions: Pfc (piriform cortex), H (hippocampus), Oc (occipital cortex), Tc (temporal cortex), Pc (parietal cortex), Fc (frontal cortex), S (striatum), T (thalamus), HT (hypothalamus), M (mesencephalon), Mob (medulla oblongata), Cm (cerebellar nuclei), Cv (cerebellar vermis), Cc (cerebellar cortex).

**REFERENCES**

1. Wenborn A, Terry C, Gros N, et al. A novel and rapid method for obtaining high titre intact prion strains from mammalian brain. Sci Rep [online serial]. 2015;5:10062. Accessed at: <http://pubmed.gov/25950908>.

2. Vidal E, Sánchez-Martín MA, Eraña H, et al. Bona fide atypical scrapie faithfully reproduced for the first time in a rodent model. Acta Neuropathol Commun. 2022;10:179.

3. Vidal E, Eraña H, Charco JM, et al. Conservation of strain properties of bank vole-adapted chronic wasting disease in the absence of glycosylation and membrane anchoring. Neurobiol Dis. 2025;210:106894.
